# Mathematical model studies of the comprehensive generation of major and minor phyllotactic patterns in plants with a predominant focus on orixate phyllotaxis

**DOI:** 10.1371/journal.pcbi.1007044

**Published:** 2019-06-06

**Authors:** Takaaki Yonekura, Akitoshi Iwamoto, Hironori Fujita, Munetaka Sugiyama

**Affiliations:** 1 Botanical Gardens, Graduate School of Science, The University of Tokyo, Bunkyo-ku, Tokyo, Japan; 2 Department of Biology, Tokyo Gakugei University, Koganei, Tokyo, Japan; 3 Astrobiology Center, National Institutes of Natural Sciences, Mitaka, Tokyo, Japan; 4 National Institute for Basic Biology, National Institutes of Natural Sciences, Okazaki, Aichi, Japan; Purdue University, UNITED STATES

## Abstract

Plant leaves are arranged around the stem in a beautiful geometry that is called phyllotaxis. In the majority of plants, phyllotaxis exhibits a distichous, Fibonacci spiral, decussate, or tricussate pattern. To explain the regularity and limited variety of phyllotactic patterns, many theoretical models have been proposed, mostly based on the notion that a repulsive interaction between leaf primordia determines the position of primordium initiation. Among them, particularly notable are the two models of Douady and Couder (alternate-specific form, DC1; more generalized form, DC2), the key assumptions of which are that each leaf primordium emits a constant power that inhibits new primordium formation and that this inhibitory effect decreases with distance. It was previously demonstrated by computer simulations that any major type of phyllotaxis can occur as a self-organizing stable pattern in the framework of DC models. However, several phyllotactic types remain unaddressed. An interesting example is orixate phyllotaxis, which has a tetrastichous alternate pattern with periodic repetition of a sequence of different divergence angles: 180°, 90°, −180°, and −90°. Although the term orixate phyllotaxis was derived from *Orixa japonica*, this type is observed in several distant taxa, suggesting that it may reflect some aspects of a common mechanism of phyllotactic patterning. Here we examined DC models regarding the ability to produce orixate phyllotaxis and found that model expansion via the introduction of primordial age-dependent changes of the inhibitory power is absolutely necessary for the establishment of orixate phyllotaxis. The orixate patterns generated by the expanded version of DC2 (EDC2) were shown to share morphological details with real orixate phyllotaxis. Furthermore, the simulation results obtained using EDC2 fitted better the natural distribution of phyllotactic patterns than did those obtained using the previous models. Our findings imply that changing the inhibitory power is generally an important component of the phyllotactic patterning mechanism.

## Author summary

Phyllotaxis, the beautiful geometry of plant-leaf arrangement around the stem, has long attracted the attention of researchers of biological-pattern formation. Many mathematical models, as typified by those of Douady and Couder (alternate-specific form, DC1; more generalized form, DC2), have been proposed for phyllotactic patterning, mostly based on the notion that a repulsive interaction between leaf primordia spatially regulates primordium initiation. In the framework of DC models, which assume that each primordium emits a constant power that inhibits new primordium formation and that this inhibitory effect decreases with distance, the major types (but not all types) of phyllotaxis can occur as stable patterns. Orixate phyllotaxis, which has a tetrastichous alternate pattern with a four-cycle sequence of the divergence angle, is an interesting example of an unaddressed phyllotaxis type. Here, we examined DC models regarding the ability to produce orixate phyllotaxis and found that model expansion by introducing primordial age-dependent changes of the inhibitory power is absolutely necessary for the establishment of orixate phyllotaxis. The simulation results obtained using the expanded version of DC2 (EDC2) fitted well the natural distribution of phyllotactic patterns. Our findings imply that changing the inhibitory power is generally an important component of the phyllotactic patterning mechanism.

## Introduction

Plants bear leaves around the stem in a regular arrangement; this is termed phyllotaxis. Across diverse plant species, phyllotaxis has common characteristics, which are often described mathematically and are reflected in a limited variety of phyllotactic patterns, including the distichous, decussate, tricussate, and Fibonacci spiral (spiral with a divergence angle close to the golden angle of 137.5°) patterns [1].

The origin of the regularity of, and the few particular patterns that are allowed in, phyllotaxis have long been fascinating questions for botanists. In the early days, morphological studies attributed phyllotactic patterning to Hofmeister’s axiom, which claims that, on the periphery of the shoot apical meristem (SAM), a new leaf primordium is formed in the largest gap between existing primordia and as far away as possible from them [2]. Following this axiom, many theoretical models have been proposed to explain the generation of phyllotactic patterns [3–21]. Such theoretical models are based on a common concept: the existence of an inhibitory field created by a repulsive, either physical or chemical, interaction between leaf primordia, which conforms to Hofmeister’s axiom. Among them, the two mathematical models proposed by Douady and Couder [15–18] are particularly notable (they will be referred to as DC1 and DC2 hereafter). The key assumptions shared by DC models are that each individual leaf primordium emits a constant power that inhibits the production of a new primordium near it and that the inhibitory effect of this power decreases as the distance from the emission point increases. In DC1, it is additionally assumed that leaf primordia are formed one by one at a constant time interval, i.e., plastochron; thus, DC1 deals only with alternate phyllotaxis [15, 16]. In contrast, DC2 does not deny the simultaneous formation of leaf primordia or temporal changes of the plastochron and can deal with both alternate and whorled phyllotaxis [17]. Computer simulations using DC models demonstrated that they can generate various major standard phyllotactic patterns as stable patterns that depend on parameter settings [15–17].

In the early 2000s, experimental studies showed that auxin determines the initiation of shoot lateral organs and that its polar transport serves as a driving force of phyllotactic patterning [22–24]. Briefly, the auxin efflux carrier PIN1, which is localized asymmetrically in epidermal cells of the shoot apex, polarly transports auxin to create auxin convergence, thus directing the position of lateral organ initiation. Subsequently, assuming the existence of a positive feedback regulatory loop between the auxin concentration gradient and PIN1 localization, a novel mathematical model was developed to explain the spontaneous formation of the auxin convergence. It was further shown by computer simulation analysis that these models can produce several typical patterns of standard phyllotaxis [25, 26]. In the auxin-transport-based models, auxin polar transport toward the auxin convergence removes auxin from its surroundings, which prevents the formation of a new, vicinal auxin convergence. This effect is considered to correspond to the repulsive interaction between primordia described in the previous models. The parameters of the auxin-transport-based model were mapped on the parameters of DC2 [27, 28], which shows that DC2 can be treated as an abstract model of the auxin-transport-based models.

**Fig 1:**
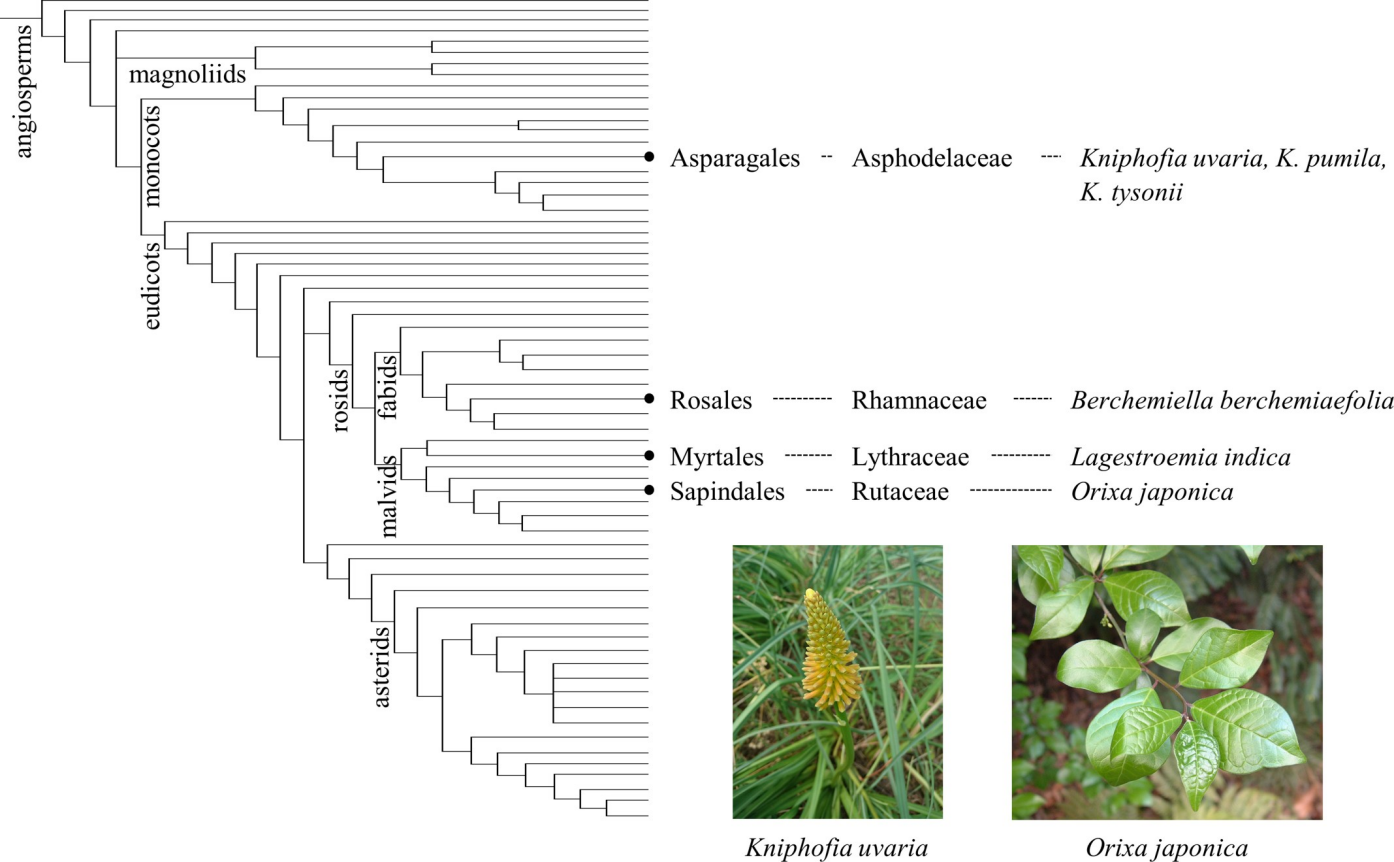
Occurrence of plants with orixate phyllotaxis in the angiosperm phylogeny. Plants with orixate phyllotaxis and their positions in the order-level phylogenetic tree of angiosperms based on Angiosperm Phylogeny Poster [33].

DC models and the auxin-transport-based models, DC2 in particular, have been studied extensively regarding the ability to produce the various phyllotactic patterns that are observed in nature [15–17, 25–26]; however, several types were never addressed in the studies that used these models. An interesting example is orixate phyllotaxis, which is named after *Orixa japonica* (Rutaceae, Sapindales) [29]. Orixate phyllotaxis is a tetrastichous alternate phyllotaxis that is characterized by the periodic repetition of a sequence of different divergence angles: 180°, 90°, −180° (180°), and −90° (270°). Although plant species that show orixate phyllotaxis are uncommon, they are found in several distant taxa (Fig 1). Many species of *Kniphofia* (Asphodelaceae, Asparagales) display a tetrastichous arrangement of leaves [30], and *K*. *uvaria*, *K*. *pumila*, and *K*. *tysonii* exhibit orixate phyllotaxis [31, 32]. *Lagestroemia indica* (Lythraceae, Myrtales) and *Berchemiella berchemiaefolia* (Rhamnaceae, Rosales) are also known as species with orixate phyllotaxis [29]. The rare and sporadic distribution of orixate phyllotaxis among plants suggests that this peculiar phyllotaxis occurred independently a few times during plant evolution. Therefore, it is likely that orixate phyllotaxis is generated by a common regulatory mechanism of leaf-primordium formation under some particular condition rather than by an orixate-unique mechanism. If this is true, mathematical models that account fully for the spatial regulation of leaf-primordium formation should be able to produce not only major phyllotactic patterns, but also orixate phyllotaxis.

In this study, we re-examined the original DC models exhaustively under various parameter conditions, to test whether they can produce orixate phyllotaxis. We then expanded DC models by introducing primordial age-dependent changes in the inhibitory power. Our results indicate that a late and slow increase in the inhibitory power is critical for the establishment of orixate phyllotaxis and imply that changing the inhibitory power is generally an important component of the mechanism of phyllotactic patterning.

## Material, methods, and models

### Plant material

Terminal winter buds of *O*. *japonica* that had been collected in July from nine plants growing at the Koishikawa Botanical Gardens, Graduate School of Science, The University of Tokyo were used for morphological analyses.

### Microscopic observation of winter buds

The winter buds were fixed with 5% v/v formalin, 5% v/v acetic acid, 50% v/v ethanol (FAA), dehydrated in an ethanol series, and finally infiltrated in 100% ethanol. For light microscopic observation, the dehydrated samples were embedded in Technovit 7100, cut into 5-μm-thick sections using a rotary microtome, and stained with 0.5% w/v toluidine blue. The center of gravity was determined for each leaf primordium on the section with ImageJ (https://imagej.nih.gov) and was used as its position when measuring morphometric data.

For scanning electron microscopy (SEM), the dehydrated samples were infiltrated once with a 1:1 v/v mixture of ethanol and isoamyl acetate and twice with isoamyl acetate. Subsequently, the samples were critical point dried, sputter coated with gold–palladium, and observed using SEM (Hitachi S-3400N).

### DC1 model

The essential points of the DC1 model are as follows [15, 16].

**Fig 2:**
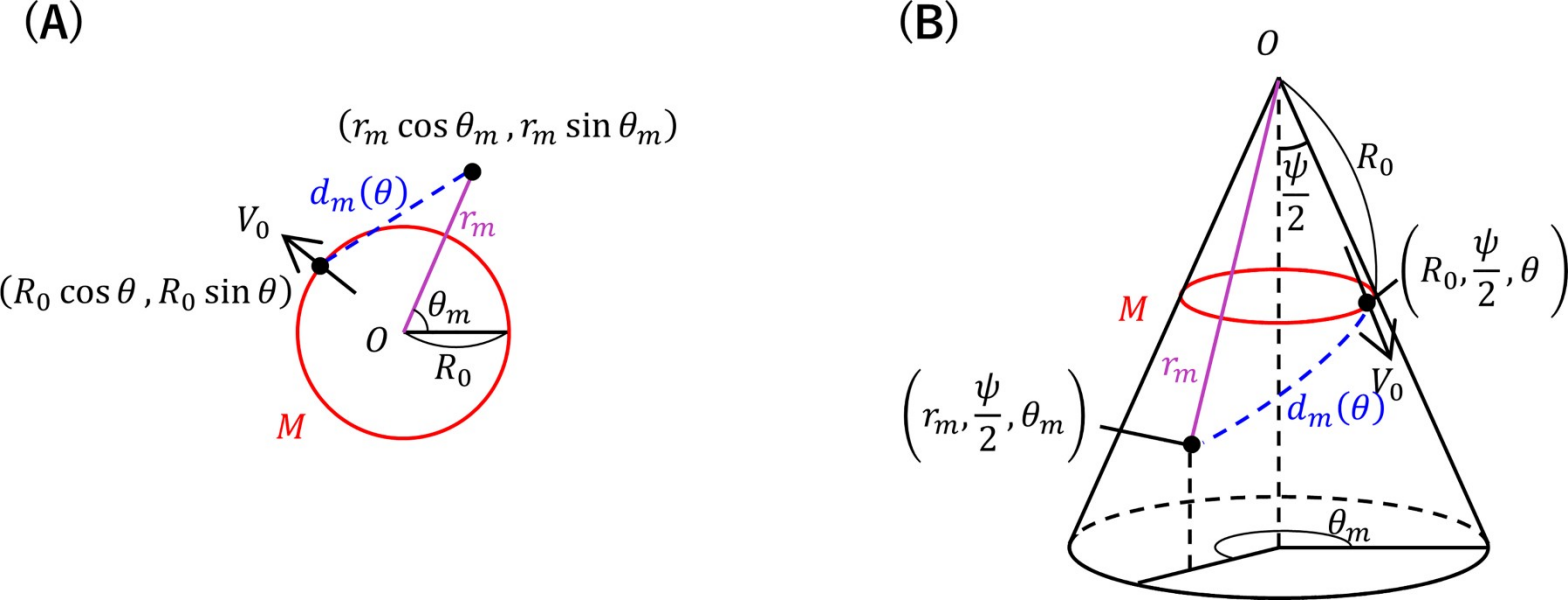
Schematic views of the shoot apex with coordinates in DC models. The shoot apex is considered as a plane in DC1 (A) and as a cone in DC2 (B).

1. The shoot apex is considered as a plane.
2. Each leaf primordium *L* emits a constant level of an inhibitory power, which generates an inhibitory field around it.
3. The inhibitory field strength decreases as a function of the distance, *d*.
4. Formation of new primordia is restricted to the SAM periphery represented by the circle *M* with a radius *R*_0_ at the shoot apex (Fig 2A).
5. New primordia are formed one by one at a regular time interval, *T*.
6. The point on *M* at which the inhibitory field strength is smallest gives the radial position of the formation of a new primordium.
7. Primordia move away from the center of the shoot apex with a radial velocity of *V*(*r*) that is proportional to the radial distance *r* because of the exponential growth of the shoot apex.

At the time when the *n*^th^ primordium *L*_*n*_ is arising, for a position (*R*_0_ cos *θ*,*R*_0_ sin *θ*) on the circle *M*, the inhibitory field strength *I*(*θ*) is calculated by summing the inhibitory effects from all preceding primordia, *L*_1_ to *L*_*n*−1_, as follows:

$$I(\theta )\equiv \sum_{m=1}^{n-1}k{\left(d_{m}(\theta \right))}^{-\eta }=k\sum_{m=1}^{n-1}{\left(R_{0}^{2}+r_{m}^{2}-2R_{0}r_{m}\;\cos\left(\theta -\theta_{m}\right)\right)}^{-\frac{\eta }{2}},$$ (1)

where *d*_*m*_ is the distance between the position (*R*_0_ cos *θ*, *R*_0_ sin *θ*) and the *m*^th^ primordium (*r*_*m*_ cos *θ*_*m*_,*r*_*m*_ sin *θ*_*m*_) and *k* is a proportional coefficient (Fig 2A). In this equation, the inhibitory field strength is assumed to be inversely proportional to the *η*^th^ power of the distance from the point emitting the inhibitory power.

Considering assumptions 5 and 7, the distance from the center of the shoot apex to the *m*^th^ primordium (*r*_*m*_) is expressed with the initial radial velocity *V*_0_ as:

$$r_{m}=R_{0}e^{\frac{V_{0}}{R_{0}}(n-m)T}.$$ (2)

The total inhibitory field strength *I* is expressed as:

$$I(\theta )=\frac{k}{R_{0}^{\eta }}\sum_{m=1}^{n-1}{\left\{1+e^{2(n-m)G}-2e^{(n-m)G}\cos\left(\theta -\theta_{m}\right)\right\}}^{-\frac{\eta }{2}},$$ (3)

where *G* is defined as *G*≡*V*_0_*T*/*R*_0_ = ln(*r*_*m*_/*r*_*m+1*_). Morphometrically, *r*_*m*_/*r*_*m+1*_ is identical to the “plastochron ratio” introduced by Richards [34].

The point (*R*_0_ cos *θ*, *R*_0_ sin *θ*) where *I*(*θ*) is smallest is chosen for the position of a new primordium. Note that *η* and *G* are the only relevant parameters that influence the behavior of *I*(*θ*) in DC1.

### DC2 model

The essential points of the DC2 model are as follows [17].

1. The shoot apex is considered as a cone with an apical angle of *ψ*.
2. Each leaf primordium *L* emits a constant level of an inhibitory power, which generates an inhibitory field around it.
3. The inhibitory field strength decreases as a function of the distance, *d*.
4. The formation of new primordia is restricted to the SAM periphery represented by the circle *M* with a distance of *R*_0_ from the conical vertex.
5. When the inhibitory field strength falls below a given threshold *E*_*s*_ somewhere on *M*, a new primordium is formed immediately at that point.
6. Primordia move away from the center of the shoot apex with a radial velocity of *V*(*r*) that is proportional to the radial distance *r* because of the exponential growth of the shoot apex.

Positions on the conical surface are expressed in spherical coordinates $\left(r,\frac{\psi }{2},\theta \right)$ (Fig 2B). The inhibitory field strength *I*(*θ*) at the position $\left(R_{0},\frac{\psi }{2},\theta \right)$ on *M* is calculated by summing the inhibitory effects from all preceding primordia, *L*_1_ to *L*_*n*−1_, as follows:

$$I(\theta )\equiv \sum_{m=1}^{n-1}E(\frac{d_{m}(\theta )}{d_{0}}),$$ (4)

where *d*_*m*_ is the distance between the *m*^th^ primordium and the position $(R_{0},\frac{\psi }{2},\theta ),$ *d*_0_ is the maximum distance within which an existing primordium excludes a new primordium, and *E* is the inhibitory effect from the preceding primordium, which is defined as a monotonically decreasing, downward-convex function:

$$E(x)\equiv E_{s}\frac{{-1+(\tanh\;\alpha x)}^{-1}}{{-1+(\tanh\alpha )}^{-1}},$$ (5)

where, if *I*(*θ*)<*E*_*s*_, a new primordium is placed at the position $\left(R_{0},\frac{\psi }{2},\theta \right)$. Throughout this study, *E*_*s*_ = 1.

Because of assumption 6, the distance from the center of the shoot apex to the *m*^th^ primordium on the conical surface (*r*_*m*_) is expressed with the time after its emergence *T*_*m*_ and the initial radial velocity *V*_0_ as:

$$r_{m}=R_{0}e^{\frac{V_{0}}{R_{0}}T_{m}}.$$ (6)

By using *t*_*m*_≡*T*_*m*_*V*_0_/*R*_0_, a standardized age of the *m*^th^ primordium defined as the product of *T*_*m*_ and the relative SAM growth rate *V*_0_/*R*_0_, *r*_*m*_ is more simply expressed as:

$$r_{m}=R_{0}e^{t_{m}}.$$ (7)

The DC2 model is characterized by three parameters: *α*, $N\equiv \sin\frac{\psi }{2}$, and $\Gamma \equiv \frac{d_{0}}{R_{0}\sqrt{N}}$. These parameters represent the steepness of the decline of the inhibitory effect around the threshold, the flatness of the shoot apex, and the ratio of the inhibition range to the SAM size, respectively.

In DC2, as a distance between points $\left(r_{\left(1\right)},\frac{\psi }{2},\theta_{\left(1\right)}\right)$ and $\left(r_{\left(2\right)},\frac{\psi }{2},\theta_{\left(2\right)}\right)$ on the conical surface, instead of the true Euclidian distance, its slightly modified version (as defined in the following equation) was used to avoid the discontinuity problem [17]:

$$d\equiv \sqrt{\frac{{\left(r_{\left(1\right)}-r_{\left(2\right)}\right)}^{2}}{N}+2Nr_{\left(1\right)}r_{\left(2\right)}\left\{1-\cos\left(\theta_{\left(1\right)}-\theta_{\left(2\right)}\right)\right\}}.$$ (8)

### Computer simulation

Model simulations were implemented in C++ with Visual C++ in Microsoft Visual Studio 2015 as an integrated development environment. Contour mapping was performed using OpenCV ver. 3.3.1 (https://opencv.org/).

Computer simulations using DC2 and DC2-derived models were initiated by placing a single primordium or two primordia at a central angle of 120° on the SAM periphery. In the former initial condition, the second primordium arises at a certain time or immediately after the first primordium, in dependence on parameter settings, at the opposite position, and in some cases, more primordia are immediately inserted at middle positions. Thus computer simulations with this condition substantially cover situations starting with 1×2^*x*^ primordia (*x* = 0,1,2⋯) evenly distributed on the SAM periphery. Similarly, simulations with the latter condition substantially cover situations starting with 3×2^*x*^ primordia (*x* = 0,1,2⋯). We also tested simulations with another initial condition, in which two primordia were placed at opposite positions with a central angle of 180°, but they returned completely same results as simulations initiated by placing a single primordium did and are therefore omitted.

Computer simulations were performed with an angle resolution of 0.1°. DC2 and DC2-derived models were simulated with a time step of Δ*t*_*m*_ = 0.001.

**Fig 3:**
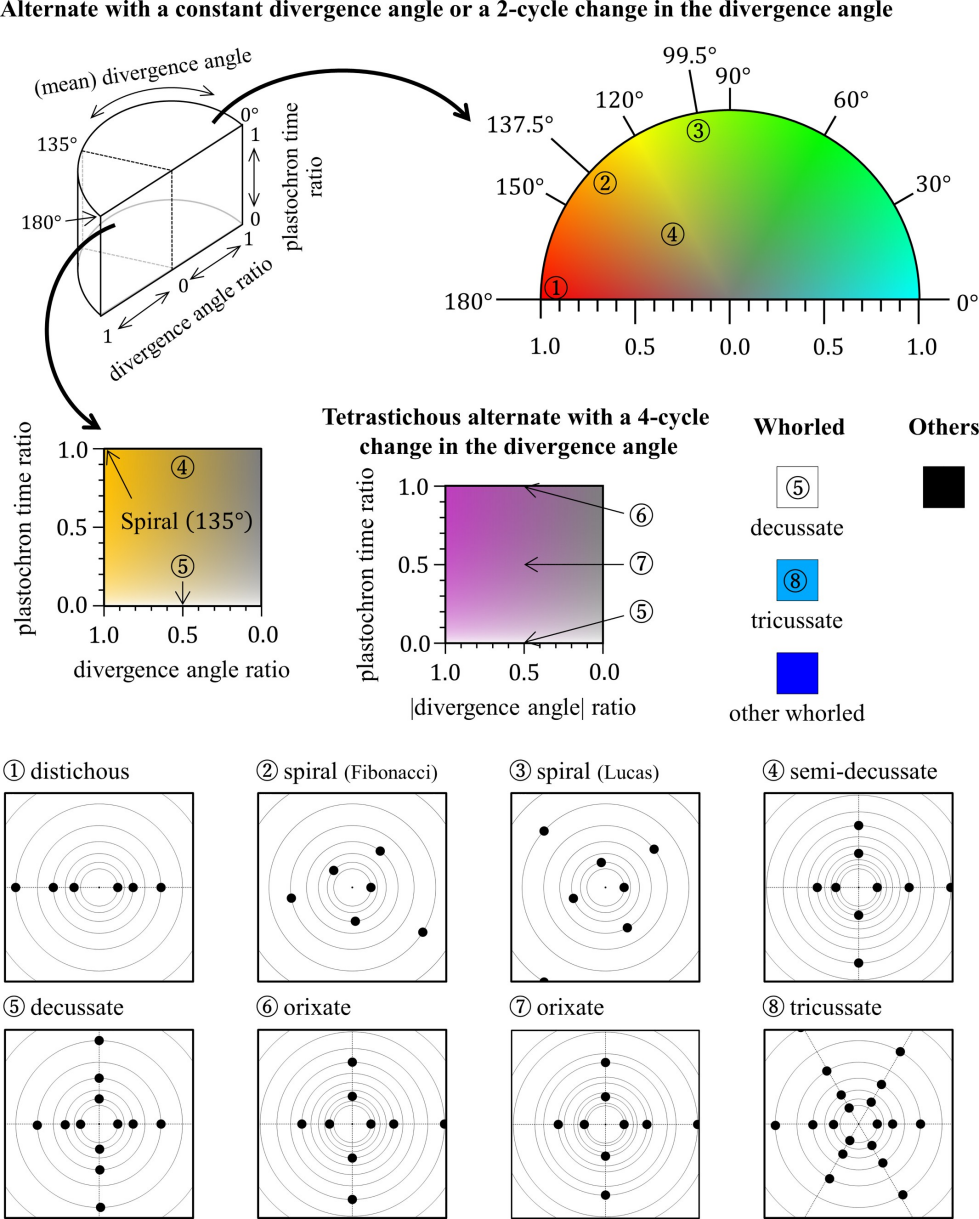
Color legend for the phyllotactic patterns generated in computer simulations. The phyllotactic patterns generated in computer simulations were classified into an alternate pattern with a constant divergence angle or a two-cycle change in the divergence angle; a tetrastichous alternate pattern with a four-cycle change in the divergence angle; a whorled pattern; and other patterns. Whorled patterns were further classified into decussate (“opposite phyllotaxis” typified by true decussate), tricussate, and other whorled patterns. These patterns were distinguished using different colors. For regular alternate patterns with a constant divergence angle, the divergence angle was indicated by a color hue from cyan (0°) to red (180°). In the case of alternate patterns with a two-cycle divergence angle change, the color hue was assigned for the mean value of the successive divergence angles. In these two-cycle alternate patterns, small-to-large ratios of two successive plastochron times and two successive divergence angles were represented by lightness (full lightness for 0) and saturation (full saturation for 1), respectively. Tetrastichous alternate patterns with a four-cycle divergence angle change were similarly expressed by color brightness and saturation based on their ratios of plastochron times and divergence angles; however, instead of the divergence angles themselves, the absolute values of divergence angles were used to calculate the ratio of divergence angles. As the divergence angle of this type of alternate pattern changes in the sequence of *p*, *q*, −*p*, and −*q* (−180°<*p*,*q*≤180°), |*q*|/|*p*| gives the ratio of the absolute values of divergence angles if |*p*|>|*q*|. Typical examples of phyllotactic patterns are marked with circled numbers in the color legend and their schematic diagrams are shown at the bottom.

In all model simulations, calculation was iterated until the total number of primordia reached 100. For alternate patterns generated by simulation, the last nine primordia were used to judge the stability and regularity of divergence angles. For the other patterns, the last two nodes were used to judge the stability of the number of primordia per node. Then the patterns were categorized and displayed as shown in Fig 3.

## Results

### Morphological characterization of phyllotaxis in *Orixa japonica*

**Fig 4:**
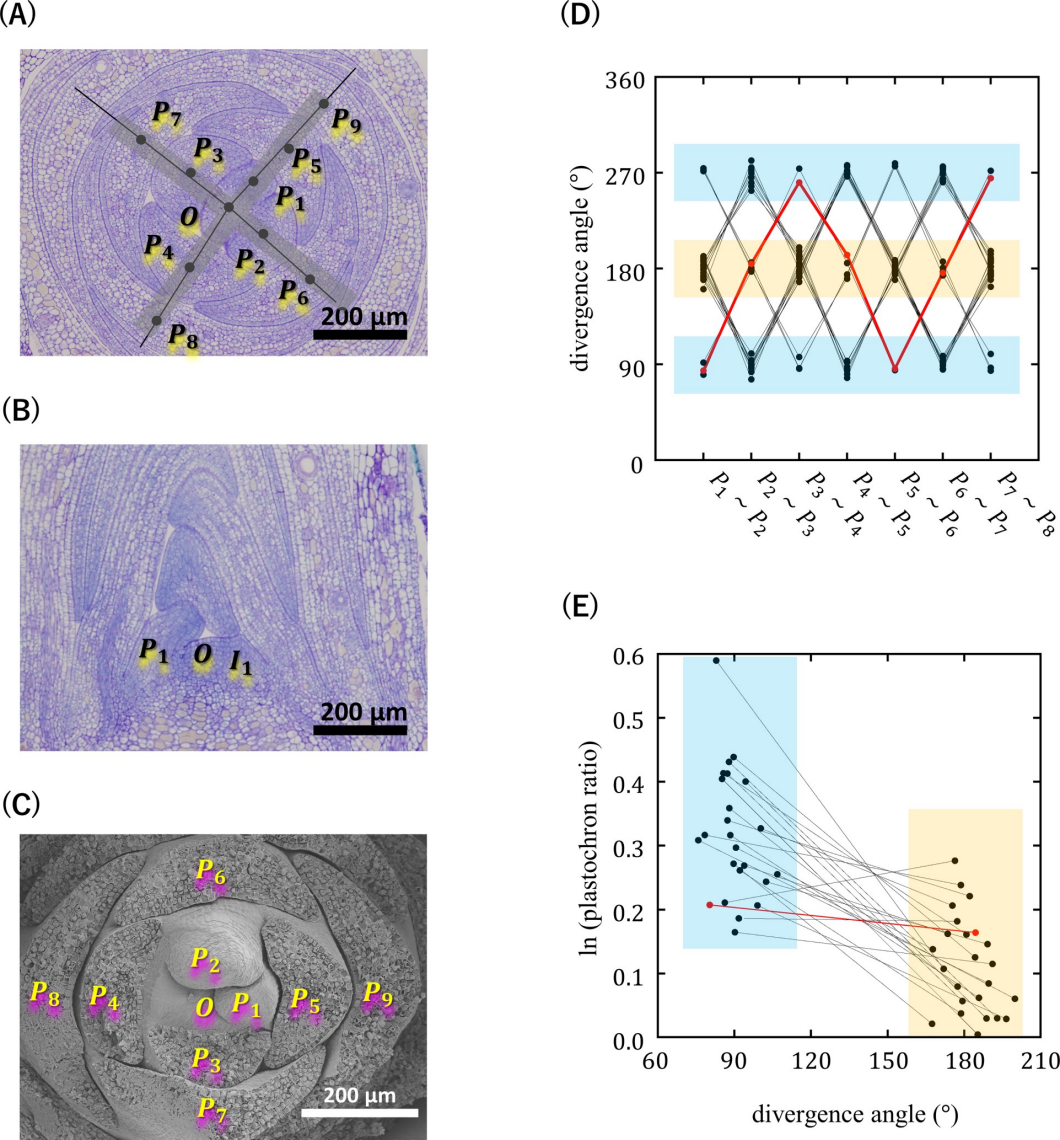
Orixate phyllotaxis in the apical winter buds of *Orixa japonica*. (A) Transverse section. *O* points to the summit of the SAM, and leaf primordia are designated as *P*_1_, *P*_2_, *P*_3_, etc., with *P*_1_ being the youngest visible primordium. Black lines represent orthostichies drawn by joining the gravity centers of leaf primorida and *O*. The four orthostichy lines can be roughly approximated by two orthogonal lines (pale gray broad lines). (B) Longitudinal section. *I*_1_ indicates the incipient primordium. (C) Scanning electron microscopic image. (D) Divergence angles measured using the transverse sections. Divergence angles close to 180° show opposite positioning of the successive primordia (blue), while angles near 90° or 270° show adjacent positioning (yellow). (E) The natural logs of plastochron ratios *OP*_2_/*OP*_1_ and *OP*_3_/*OP*_2_ are plotted based on whether the two primordia are located in an adjacent or opposite position. In (D) and (E), points linked by a line represent data from the same sample, and red points indicate data obtained from the section of (A).

First, we performed an anatomical analysis of the apical winter buds of *O*. *japonica*, to characterize morphologically its phyllotaxis. In the transverse sections of the winter buds, there was a very obvious tetrastichous pattern of leaf primordia, which were arranged in opposite pairs on either of two orthogonal lines (Fig 4A). This pattern looked similar to decussate phyllotaxis; however, unlike decussate phyllotaxis, it was not symmetric. Opposite pairs of primordia varied in size and radial distance and, in each pair, a smaller primordium was positioned closer to the center of the shoot apex. Such asymmetry was also clearly recognized in the longitudinal sections and by observations performed using SEM (Fig 4B and 4C). Importantly, SEM observations detected incipient primordia that were not paired (Fig 4C). Therefore, the asymmetric arrangement of leaves was attributed to the alternate initiation of leaf primordia instead of the secondary displacement of originally decussate leaf primordia. The divergence angle between successive primordia changed in the sequence of approximately 180°, 90°, −180° (180°), and −90° (270°), and this cycle was repeated a few times in the winter bud (Fig 4D). These results confirmed that the phyllotaxis of *O*. *japonica* is genuinely an “orixate phyllotaxis”.

Richards’ plastochron ratio was found to oscillate in relation to the divergence angle. Plastochron ratios measured from the adjacent pairs of primordia with a divergence angle of approximately ±90° were significantly larger than those measured from the opposite pairs with a divergence angle of approximately ±180° (Fig 4E). A similar relationship between divergence angles and plastochron ratios had been, albeit fragmentarily, described for the orixate phyllotaxis of *K*. *uvaria* [32]; thus, it is likely to be a common feature of orixate phyllotaxis.

### Computer simulation assessment of DC1 regarding the ability to produce orixate phyllotactic patterns

DC1 is an inhibitory field model specialized for alternate phyllotactic patterning. DC1 assumes one-by-one formation of leaf primordia at a constant time interval, which strongly limits the model flexibility [16]. Nevertheless, as this constraint makes the patterning process simple and possible to be dealt with theoretically, it is worth investigating DC1 as a primary model for generation of any types of alternate phyllotaxis.

**Fig 5:**
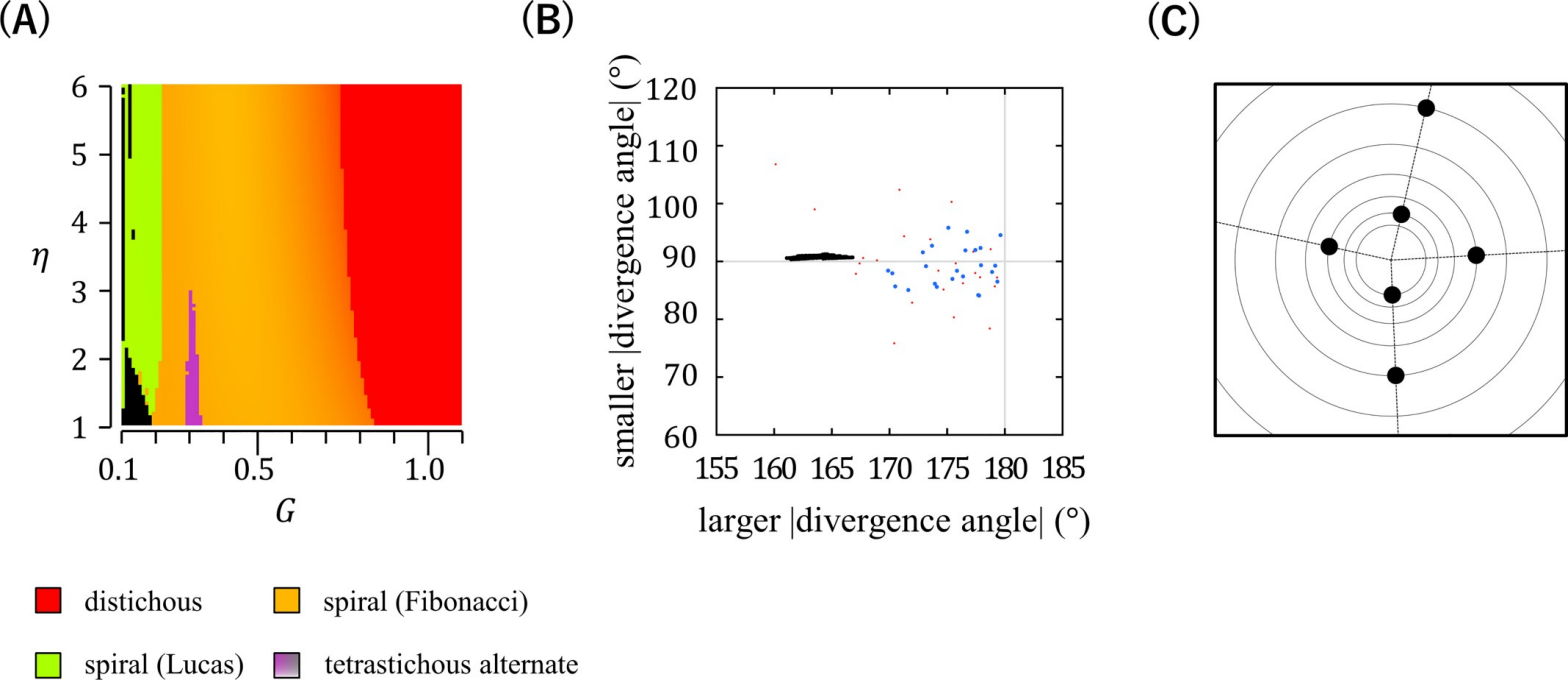
Phyllotactic patterns generated in computer simulations using DC1. (A) Computer simulations using DC1 were performed under various settings of parameters *G* and *η* (101×101 conditions), and the patterns obtained are displayed according to the color legend shown in Fig 3. (B) The black and red dots indicate the absolute values of divergence angles of the tetrastichous alternate patterns generated in (A) and real orixate phyllotaxis observed for winter buds of *O*. *japonica* (data of *P*_1_~*P*_2_ and *P*_2_~*P*_3_ in Fig 4D), respectively. The blue dots show the averages determined from the real data of *P*_1_~*P*_2_ to *P*_6_~*P*_7_ (Fig 4D) for each winter bud of *O*. *japonica*. In this panel, alternate patterns with a four-cycle change in divergence angles in the sequence of *p*, *q*, −*p*, and −*q* (|*p*|>|*q*|) were plotted at the point (|*p*|,|*q*|). (C) An example of the tetrastichous alternate patterns, which was produced by computer simulation at *G* = 0.3 and *η* = 1.5. This pattern has a divergence angle change in the sequence of 165°, −91°, −165°, and 91° and, unlike orixate phyllotaxis, exhibits a distorted tetrastichy, rather than an orthogonal tetrastichy.

To test whether DC1 can produce orixate phyllotaxis, we re-examined this established model via detailed computer simulation analysis using exhaustive combinations of the determinant parameters, *η* and *G*. As reported previously [15, 16], distichous and relatively major spiral phyllotactic patterns, i.e., alternate patterns with a regular divergence angle near 180°, a Fibonacci angle (137.5°), or a Lucas angle (99.5°), were generated as stable patterns over broad ranges of *η* and *G* in these simulations (Fig 5). Of note, when *η* and *G* were set to 1–3 and about 0.2, respectively, tetrastichous patterns were formed that resembled orixate phyllotaxis, as they showed a four-cycle periodic change of the divergence angle in the order of *p*, *q*, −*p*, and −*q* (−180°≤*p*≤180°,|*p*|>|*q*|) (Fig 5A). In these patterns, however, the larger absolute value of the divergence angle was considerably deviated from 180°, whereas this should be very close to 180° in orixate phyllotaxis (Fig 5B). These patterns showed nonorthogonal tetrastichy, which is distinct in appearance from the orthogonal tetrastichy of orixate phyllotaxis (Fig 5C). Therefore, we concluded that the tetrastichous patterns found in simulations with DC1 are not orixate and that DC1 does not generate the orixate phyllotactic pattern at any parameter setting. The absence of the occurrence of normal orixate phyllotaxis, the divergence angles of which are exactly ±180° and ±90°, in the context of DC1 can be explained analytically (S1 Text).

### Expansion of DC1 by introducing age-dependent changes in the inhibitory power

Next, we examined whether modification of DC1 could enable it to produce orixate phyllotaxis. In an attempt to modify DC1, we focused on the inhibitory power of each leaf primordium against new primordium formation—which is assumed to be constant in DC models but may possibly change during leaf development—and expanded DC1 by introducing age-dependent, sigmoidal changes in the inhibitory power. In this expanded version of DC1 (EDC1), the inhibitory field strength *I*(*θ*) was redefined as the summation of the products of the age-dependent change in the inhibitory power and the distance-dependent decline of its effect:

$$I(\theta )\equiv \sum_{m=1}^{n-1}\left\{k{\left(d_{m}(\theta )\right)}^{-\eta }F(n-m)\right\}.$$ (9)

*F* is defined as:

$$F(\Delta t)\equiv \frac{1}{1+e^{-a(\Delta t-b)}},$$ (10)

where parameters *a* and *b* are constants that represent the rate and timing of the age-dependent changes in the inhibitory power, respectively. Under this equation, in an age-dependent manner, the inhibitory power increases at *a*>0 and decreases at *a*<0. In the present study, *η* was fixed at 2 for EDC1.

Prior to computer simulation analysis with EDC1, we searched for parameters of EDC1 that can fit the requirements of normal orixate phyllotaxis. When the normal pattern of orixate phyllotaxis is stably maintained, a rectangular coordinate system with the origin at the center of the shoot apex can be set such that all primordia lie on the coordinate axes, and every fourth primordium is located on the same axis in the same direction, i.e., the position of any primordium (*m*^th^ primordium) can be expressed as (*r*_*m*_ cos *θ*_*m−*4*i*_, *r*_*m*_ sin *θ*_*m*−4*i*_) for integers *i*. Under this condition, we considered whether a new primordium (*n*^th^ primordium) is produced at the position (*R*_0_ cos *θ*_*n−*4*i*_, *R*_0_ sin *θ*_*n*−4*i*_), to keep the normal orixate phyllotactic pattern. In EDC1, as in DC1, new primordium formation at (*R*_0_ cos *θ*_*n−*4*i*_, *R*_0_ sin *θ*_*n*−4*i*_) implies that the inhibitory field strength *I*(*θ*) on the circle *M* has a minimum at *θ*_*n*−4*i*_. For this reason, we first attempted to solve the following equation:

$${\frac{dI(\theta )}{d\theta }|}_{\theta -\theta_{n-4i}=0}=0.$$ (11)

**Fig 6:**
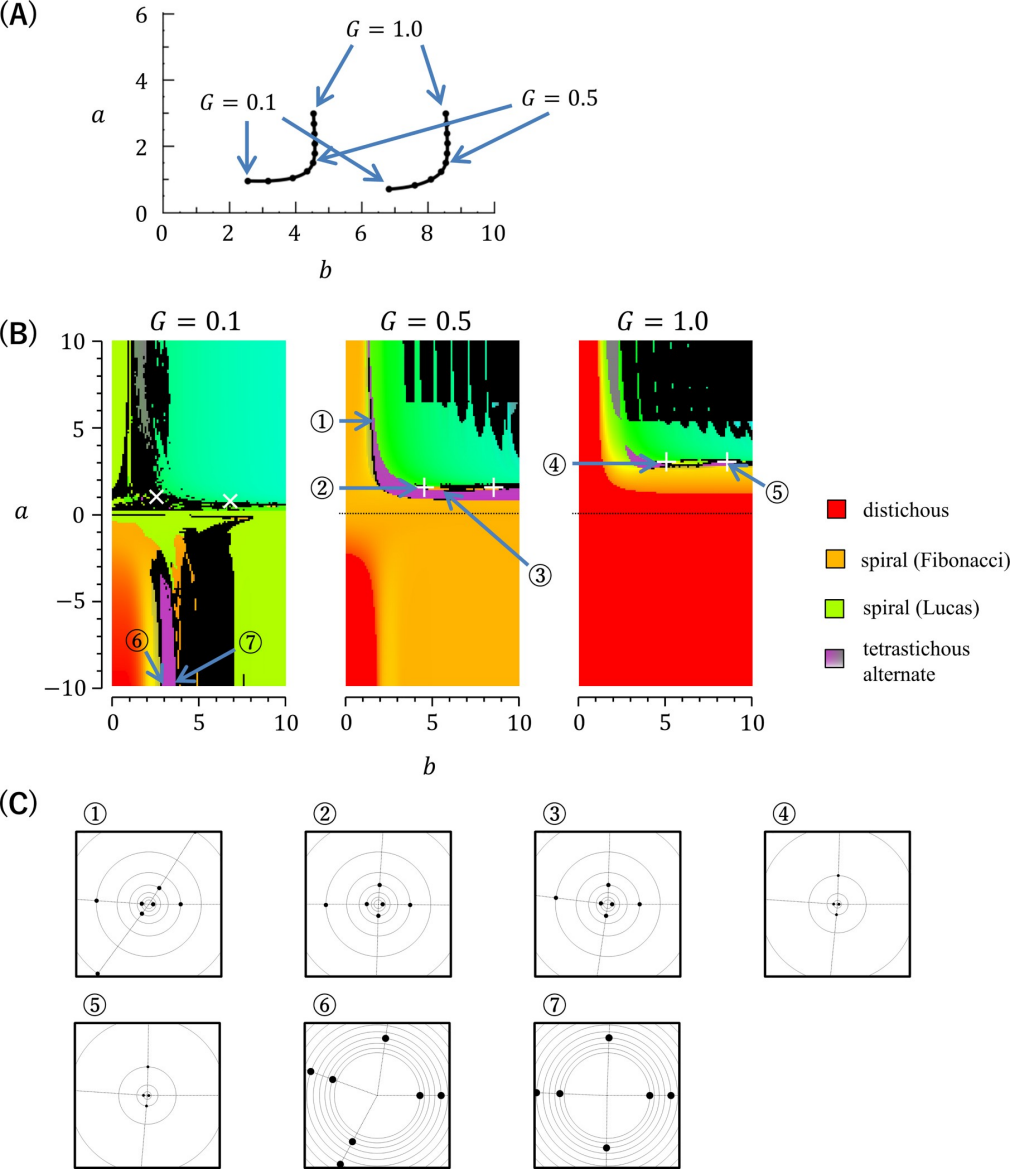
Mathematical and computer simulation analysis of EDC1. (A) Numerical solutions of parameters that fit the mathematical requirements for normal orixate phyllotaxis in EDC1. The two curves show the solutions obtained using various *G* values. The closed circles indicate the solutions obtained with *G* set at 0.1 intervals between 0.1 and 1.0. (B) Stable patterns generated in computer simulations using EDC1 under various parameter settings (201 settings for *a*, 101 settings for *b*, 3 settings for *G*, and thus 201×101×3 = 60,903 simulations in total). The patterns obtained are displayed according to the color legend shown in Fig 3. The white crosses (+) indicate the parameter conditions obtained as numerical solutions of ${\frac{dI(\theta )}{d\theta }|}_{\theta -\theta_{n-4i}=0}=0$, giving a minimum of *I*(*θ*) around *θ*_*n*−4*i*_ for normal orixate phyllotaxis, whereas white saltires (×) indicate the parameter conditions obtained as numerical solutions of ${\frac{dI(\theta )}{d\theta }|}_{\theta -\theta_{n-4i}=0}=0,$ giving a maximum of *I*(*θ*). (C) Schematic diagrams of typical examples of the phyllotactic patterns generated in the computer simulations. The circled numbers relate the diagrams to the parameter conditions shown in (B).

This equation was numerically solved under two geometrical situations of primordia: the divergence angle between the newly arising primordium and the last primordium is ±90° (situation 1) or ±180° (situation 2) (S1A Fig). The solutions obtained identified parameter sets that satisfied the above equation under both these two situations (Fig 6A, S1B Fig). The calculation of *I*(*θ*) using the identified parameter sets showed that *I*(*θ*) has a local and global minimum around *θ*_*n*−4*i*_ with large values of *G*, such as 0.5 or 1, while it has a local maximum instead of a minimum around *θ*_*n*−4*i*_ with small *G* values, such as 0.1 (S1C Fig). This result indicates the possibility that EDC1 can form orixate phyllotaxis as a stable pattern under a particular parameter setting with large *G* values.

### Generation of orixate phyllotactic patterns in a computer simulation using EDC1

We conducted computer simulations using EDC1 over broad ranges of parameters and found that EDC1 could generate tetrastichous alternate patterns in addition to distichous and spiral patterns (Fig 6B). The tetrastichous patterns included orthogonal tetrastichous ones with a four-cycle divergence angle change of approximately 180°, 90°, −180°, and −90°, which can be regarded as orixate phyllotaxis (Fig 6C, S2 Fig). Under the conditions of assuming an age-dependent increase in the inhibitory power (*a*>0), these orixate patterns were formed within a rather narrow parameter range of *G* = 0.5~1, *a* = 1~2, and *b* = 4~9 around the parameter settings that were determined by numerical solution, to fit the requirements for the stable maintenance of normal orixate phyllotaxis (Fig 6B and 6C). When assuming an age-dependent decrease in the inhibitory power (*a*<0), orixate phyllotaxis appeared at a point of *G* = 0.1, *a*≈−10, and *b*≈3.5 (Fig 6B and 6C). These values of *a* and *b* represent a very sharp drop in the inhibitory power at the primordial age corresponding to approximately three plastochron units. Around this parameter condition, there were no numerical solutions for normal orixate phyllotaxis; however, patterns that were substantially orixate, although they were not completely normal, could be established. The orixate patterns that were generated under the conditions in which the inhibitory power increased and decreased were visually characterized by sparse primordia around the small meristem and dense primordia around the large meristem, respectively (Fig 6C).

In the results of computer simulations with EDC1, besides the orixate patterns, we also found peculiar patterns with an *x*-cycle change in the divergence angle consisting of 180° followed by an (*x*−1)-times repeat of 0° (S3 Fig). Such patterns were generated when all the parameters *a*, *b*, and *G* were set to relatively large values and are displayed as periodic distribution of black regions in the upper right area of the middle and right panels of Fig 6B. In these patterns, as *b* is increased, the number of repetition times of 0° is increased, resulting in the shift from *x*-cycle to (*x*+1)-cycle. This shift is mediated by the occurrence of spiral patterns with a small divergence angle, and the transitions from *x*-cycle to spiral and from spiral to (*x*+1)-cycle takes place suddenly in response to a slight change of *b* (S3 Fig).

### Computer simulation assessment of DC2 regarding the ability of producing orixate phyllotactic patterns

**Fig 7:**
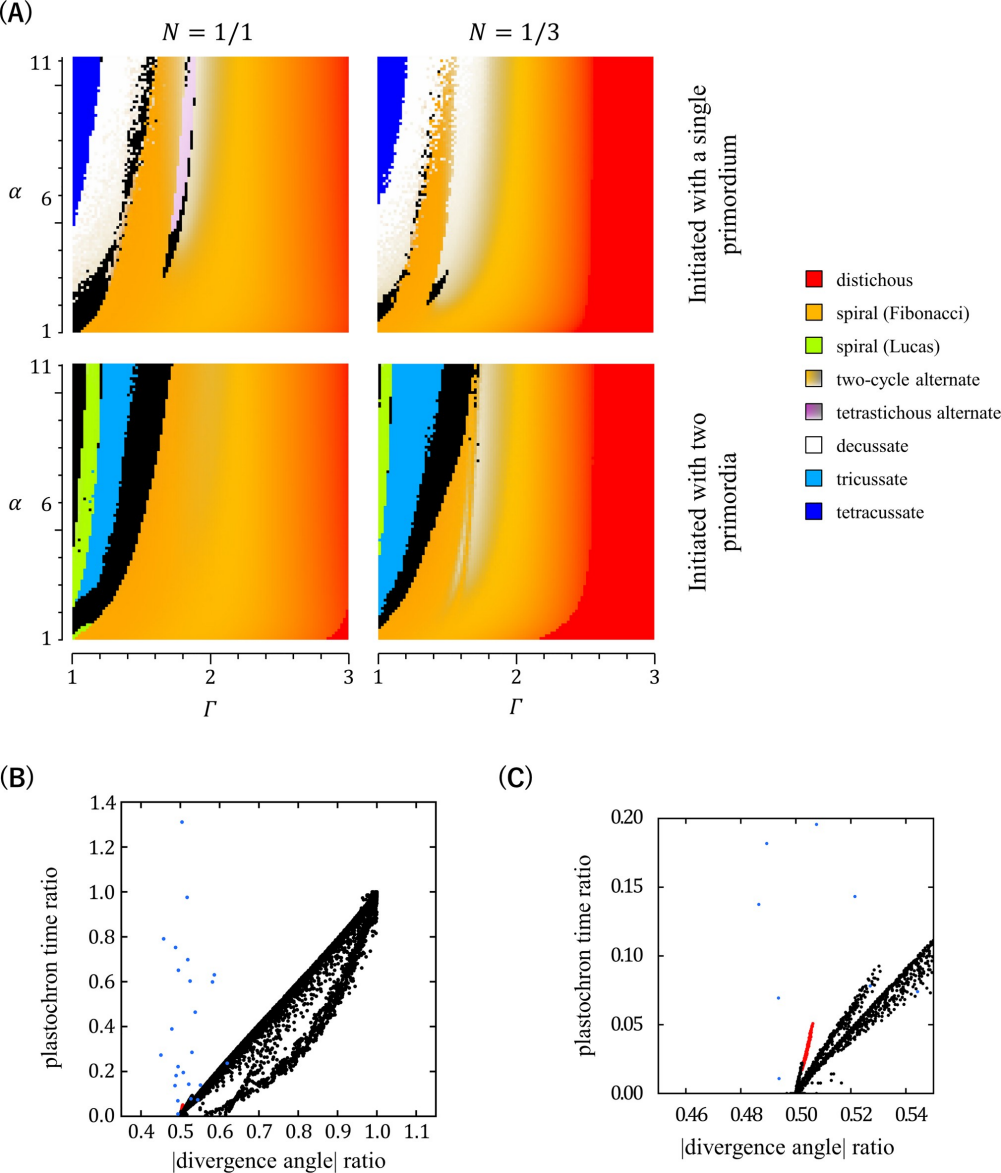
Phyllotactic patterns generated in computer simulations using DC2. (A) Computer simulations using DC2 were performed under various settings of parameters *α* and *Γ* (101 settings for *α* and 101 settings for *Γ*) with *N* = 1,1/3, or 1/5, and the resultant patterns are displayed for the cases of *N* = 1 and 1/3 according to the color legend shown in Fig 3. Simulations were started by placing a single primordium or two primordia at a central angle of 120° on the SAM periphery. (B) The regular alternate, two-cycle alternate, and tetrastichous four-cycle alternate patterns generated in computer simulations using DC2 in (A), including simulations with *N* = 1/5 as well as *N* = 1 and 1/3, were plotted using the ratio of absolute values of two successive divergence angles as the abscissa and the ratio of two successive plastochron times as the ordinate. The red dots indicate tetrastichous four-cycle patterns, while the black dots indicate regular alternate and two-cycle patterns. The blue dots show the data of real orixate phyllotaxis observed for winter buds of *O*. *japonica* (calculated from the data of *P*_1_~*P*_2_ and *P*_2_~*P*_3_ in Fig 4D). (C) Magnification of the lower-left corner of (B).

DC2, as DC1, is an inhibitory field model but is more generalized than DC1 [17]. Unlike DC1, DC2 does not assume one-by-one formation of primordia at a constant time interval and thus does not exclude whorled phyllotactic patterning. Indeed, DC2 was shown to produce all major patterns of either alternate or whorled phyllotaxis depending on parameter conditions [17]. To test whether DC2 can generate orixate phyllotactic patterns, we carried out extensive computer simulation analyses using this model. Our computer simulations confirmed that major phyllotactic patterns, such as distichous, Fibonacci spiral, Lucas spiral, decussate, and tricussate patterns, are formed as stable patterns in wide ranges of parameters, and also showed formation of tetrastichous alternate patterns with a four-cycle change of the divergence angle at *N* = 1 and *Γ*≈1.8 when initiated by placing a single primordium at the SAM periphery (Fig 7A). The possible inclusion of orixate phyllotaxis in these tetrastichous four-cycle patterns was carefully examined based on the ratio of plastochron times and the ratio of absolute values of divergence angles, which should be much larger than 0 and close to 0.5, respectively, in orixate phyllotaxis. Although all the tetrastichous four-cycle patterns detected here had a divergence angle ratio near 0.5, their ratios of plastochron times were too small to be regarded as orixate phyllotaxis, and the overall characters indicated that they are rather similar to decussate phyllotaxis (Fig 7B and 7C). These results led to the conclusion that the DC2 system does not generate orixate phyllotaxis under any parameter conditions.

### Expansion of DC2 by introducing age-dependent changes in the inhibitory power

Similar to the approach used for DC1, we expanded DC2 by introducing primordial age-dependent changes in the inhibitory power. In this expanded version of DC2 (EDC2), the inhibitory field strength *I*(*θ*) was redefined as the summation of the products of the age-dependent change in the inhibitory power and the distance-dependent decrease of its effect:

$$I(\theta )\equiv \sum_{m=1}^{n-1}\left\{E(\frac{d_{m}(\theta )}{d_{0}})F(t_{m})\right\},$$ (12)

where *F* is a function expressing a temporal change in the inhibitory power, defined as:

$$F(t)\equiv \frac{1}{1+e^{-A(t-B)}}.$$ (13)

### Generation of orixate phyllotactic patterns in a computer simulation using EDC2

**Fig 8:**
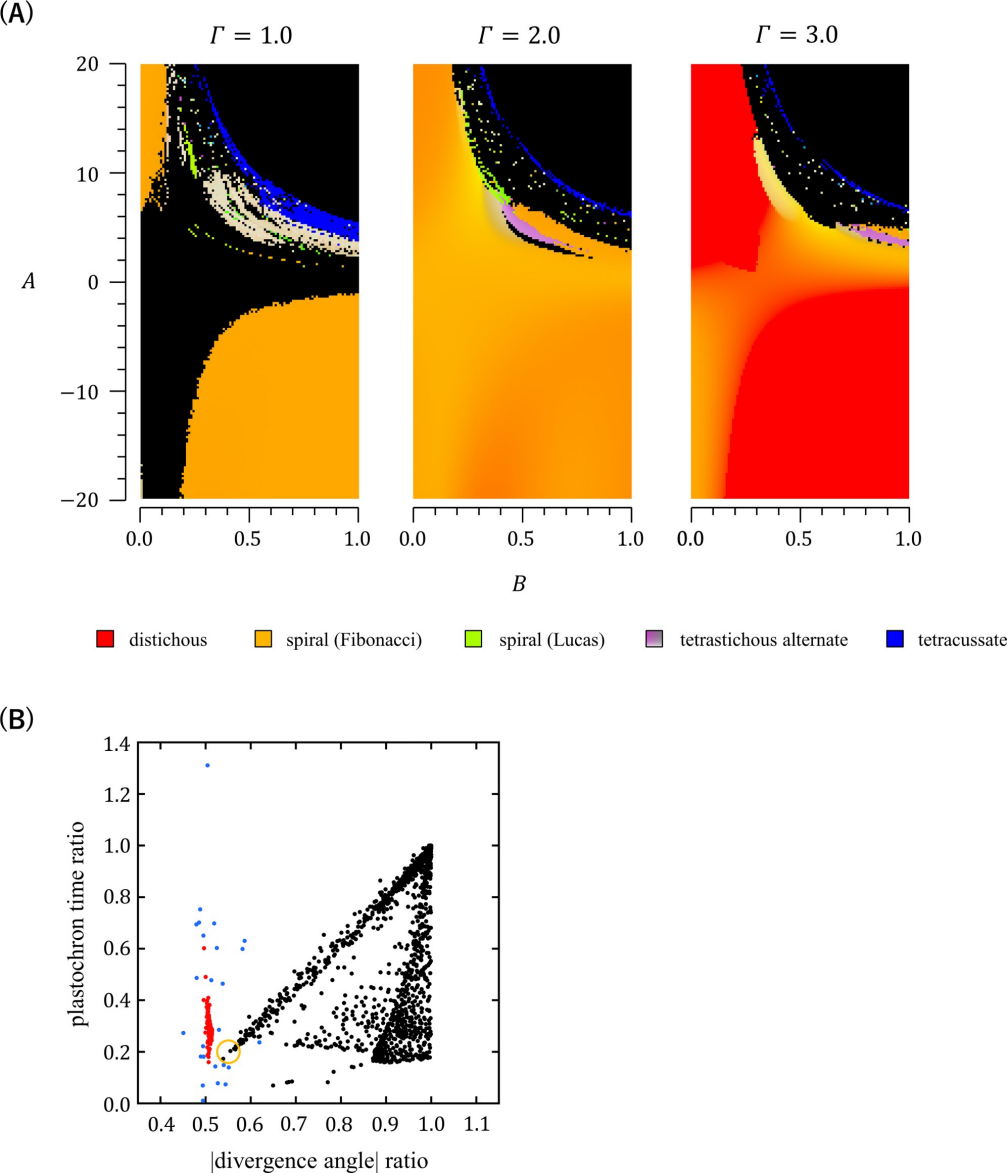
Phyllotactic patterns generated in computer simulations using EDC2. (A) Computer simulations using EDC2 were performed under various parameter settings (201 settings for −20≤*A*≤20, 101 settings for 0≤*B*≤1, and *Γ* = 1, 2, or 3) with fixed parameters *α* = 1 and *N* = 1/3, and the patterns obtained are displayed according to the color legend shown in Fig 3. Simulations were started by placing a single primordium on the SAM periphery. (B) Computer simulations using EDC2 were performed under various settings of parameters (101 settings for 0≤*A*≤20, 101 settings for 0≤*B*≤1, and *Γ* = 2, 2.5, or 3) with fixed parameters *α* = 1 and *N* = 1/3. The graph shows a scatter plot of alternate patterns with a constant divergence angle or a two-cycle change in the divergence angle (black), and tetrastichous alternate patterns with a four-cycle change in the divergence angle (red) generated in the computer simulations. In this graph, each pattern was plotted based on the ratio of absolute values of two successive divergence angles (abscissa) and the ratio of plastochron times (ordinate). The black dots surrounded by an orange circle represent semi-decussate-like patterns that occurred in the vicinities of orixate phyllotaxis in the parameter space, which are indicated by blue asterisks in S6A Fig. The blue dots indicate the data of real orixate phyllotaxis observed for winter buds of *O*. *japonica* (calculated from the data of *P*_1_~*P*_2_ and *P*_2_~*P*_3_ in Fig 4D).

**Fig 9:**
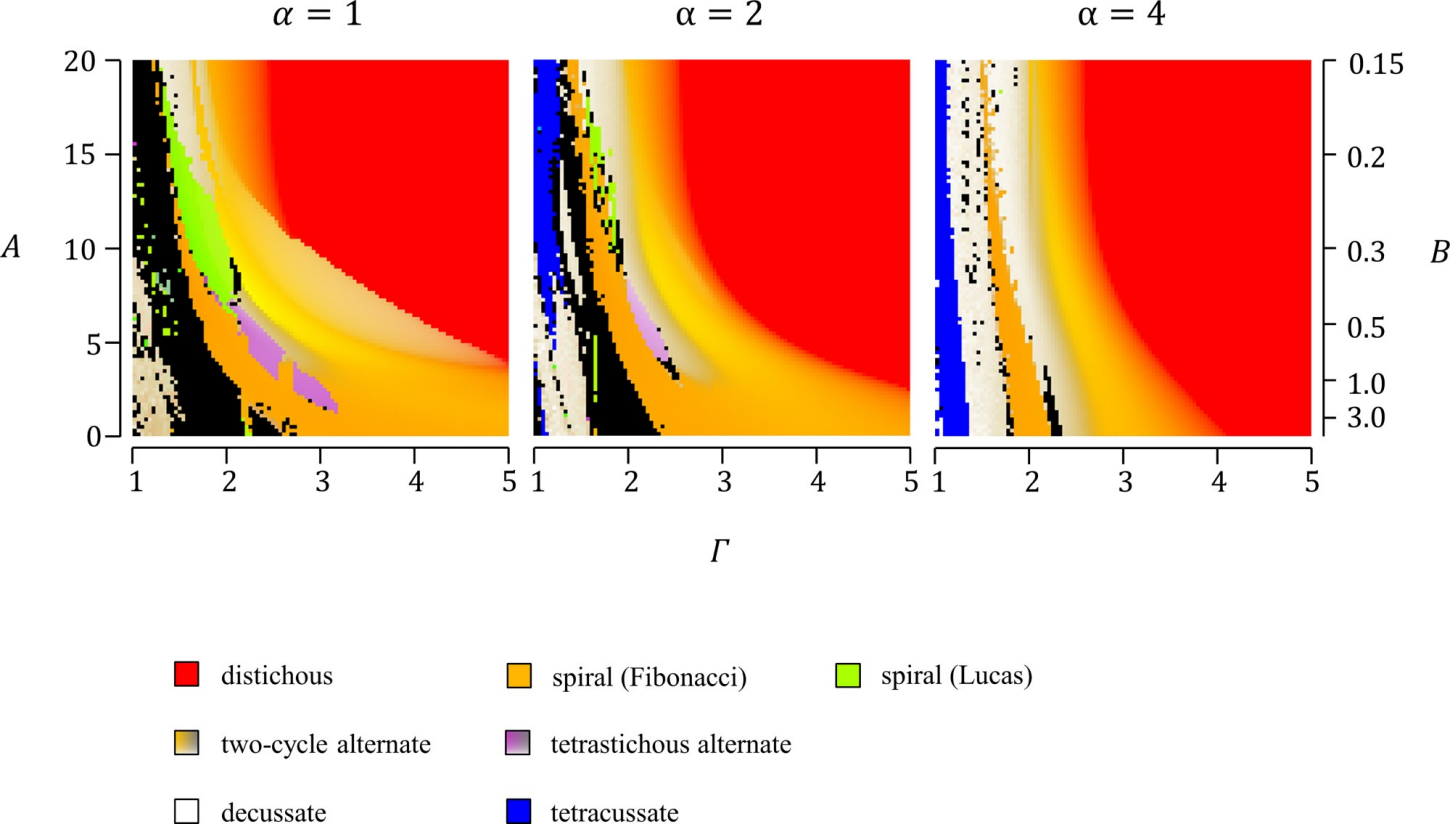
Effects of the inhibition range and increase in inhibitory power on phyllotactic patterns in EDC2. Computer simulations were performed using EDC2 with *α* = 1, 2, or 4 under various settings of *Γ* and *A* (101×101 conditions), which reflect the maximum inhibition range of a primordium and the primordial age-dependent increase in the inhibitory power, respectively. The initial value of the inhibitory power was fixed to 0.047, i.e., *A*×*B* was fixed at 3. *N* was fixed at 1/3. The simulation was started by placing a single primordium on the SAM periphery. The patterns obtained are displayed according to the color legend shown in Fig 3.

**Fig 10:**
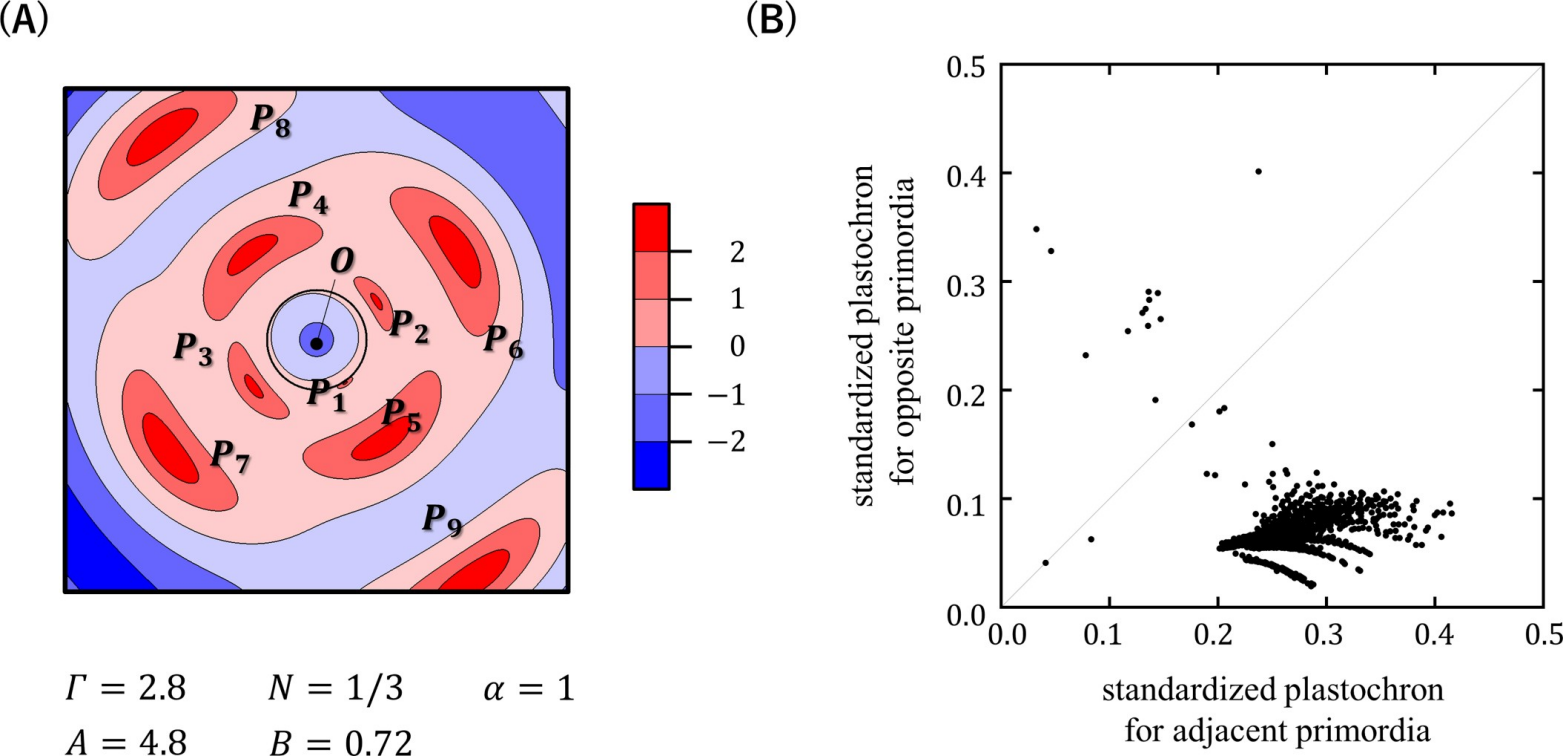
Characteristics of orixate patterns generated in computer simulations using EDC2. (A) Contour map of the natural log of the inhibitory field strength *I* within the shoot apical region that generated orixate phyllotaxis in the computer simulation using EDC2. A value of 0 implies that the inhibitory field strength is equal to the threshold for primordium formation. (B) Relationship between plastochrons and divergence angles in orixate patterns generated in computer simulations using EDC2. For a pair of successive primordia, *L*_*m*_ and *L*_*m*+1_, a standardized plastochron was calculated as *t*_*m*+1_−*t*_*m*_ = ln(*r*_*m*_/*r*_*m+1*_). Orixate patterns were plotted based on their two standardized plastochrons: one for the pair of opposite primordia with a divergence angle of approximately 180°, and the other for the pair of adjacent primordia with a divergence angle of approximately ±90°.

Computer simulations using EDC2 were first conducted under a wide range of combinations of *A* and *B* at three different settings of *Γ* (*Γ* = 1, 2, or 3) and fixed conditions for *α* and *N* (*α* = 1,*N* = 1/3) (S4 Fig). In this analysis, tetrastichous four-cycle patterns were formed within the parameter window where *A* was 3–7 and *B* was 0.4–1, which represents a late and slow increase in the inhibitory power during primordium development (Fig 8A). Further analysis performed by changing *Γ*, *α*, and *N* showed that small values of *α*, which indicate that the distance-dependent decrease in the inhibitory effect is gradual, and large values of *Γ*, which indicate that the maximum inhibition range of a primordium is large, are also important for the formation of tetrastichous four-cycle patterns (Fig 9, S5 Fig). All of these four-cycle patterns were found to be almost orthogonal and to have a sufficiently large ratio of successive plastochron times, thus fitting the criterion of orixate phyllotaxis (Fig 8B, S7 Fig). Furthermore, the plots of these patterns lied within the cloud of the data points of real orixate phyllotaxis, and therefore we concluded that they are orixate. A typical example of such orixate patterns was obtained by simulation using the parameters, *A* = 4.8, *B* = 0.72, *Γ* = 2.8, *N* = 1/3, and *α* = 1, and is presented as a contour map of the inhibitory field strength in Fig 10A, which clearly depicts orixate phyllotactic patterning. Under this parameter condition, the inhibitory field strength on the SAM periphery was calculated to have a minimum close to the threshold at 0° at the time of new primordium formation when the preceding primordia were placed at 0°, 180°, and ±90° (S8 Fig). This landscape of the inhibitory field stabilizes the orixate arrangement of primordia. In summary, our analysis demonstrated that orixate phyllotaxis comes into existence in the EDC2 system when the inhibitory power of each primordium increases at a late stage and slowly to a large maximum and when its effect decreases gradually with distance.

In the orixate phyllotactic patterns generated by EDC2, the plastochron time oscillated between two values together with a cyclic change in the divergence angle: the longer plastochron was observed for the adjacent pairs of primordia with a divergence angle of ±90° and the shorter plastochron was recorded for the opposite pairs with a divergence angle of ±180° (Fig 10B, S1 Movie). This relationship between the plastochron and the divergence angle agreed with the real linkage observed for the plastochron ratios and divergence angles in the winter buds of *O*. *japonica* (Fig 4E).

### Distribution of phyllotactic patterns in the parameter space of EDC2

Based on a comprehensive survey of the results of the computer simulations performed using EDC2, we examined the distribution of various phyllotactic patterns and the possible relationships between them in the parameter space of EDC2 (Figs 8A and 9, S4, S5, S9 and S10 Figs). Major phyllotactic patterns, such as the distichous, Fibonacci spiral, and decussate patterns, occupied large areas in the parameter space, and the Lucas spiral pattern occupied some areas. Depending on the initial condition, the tricussate pattern also took a considerable fraction of the space. In the parameter space, the distichous pattern adjoined the Fibonacci spiral pattern, while the Fibonacci spiral adjoined the distichous, Lucas spiral, decussate, and tricussate patterns. The regions where the orixate pattern was generated were located next to the regions of the decussate, Fibonacci spiral, Lucas spiral, and/or two-cycle alternate patterns. This positional relationship suggests that orixate phyllotaxis is more closely related to the decussate and spiral patterns than it is to the distichous pattern. The two-cycle patterns formed in a narrow parameter space next to the region of orixate phyllotaxis and had a divergence angle ratio of approximately 0.55 and a plastochron time ratio of approximately 0.2 (Fig 8B, S6A Fig); thus, they are similar to semi-decussate phyllotaxis, which is an alternate arrangement characterized by the oscillation of the divergence angle between 180° and 90° (S6B Fig). These semi-decussate-like patterns were not observed in the computer simulations performed using DC2 (Fig 7B and 7C); rather, they were produced only after its expansion into EDC2.

The overall distributions of major phyllotactic patterns in the parameter space were compared between DC2 and EDC2 using color plots drawn from the results of simulations conducted for EDC2 with various settings of the inhibition range parameter *Γ* and the inhibitory power change parameter *A* (Fig 9). In these simulations, large *A* values accelerated the age-dependent increase in the inhibitory power of each primordium; if *A* is sufficiently large, the inhibitory power is almost constant during primordium development and the EDC2 system is almost the same as DC2. Therefore, the colors along the top side of each panel of Fig 9, where *A* was set to 20, which is a high value, show the phyllotactic pattern distribution against *Γ* in DC2, while the colors over the two-dimensional panel show the phyllotactic pattern distribution against *Γ* and *A* in EDC2. The order of distribution of the distichous, Fibonacci spiral, and decussate patterns was unaffected by decreasing *A* and, thus, did not differ between DC2 and EDC2. As reported in the previous study of DC2 [17], on the top side of Fig 9, the stable pattern changed from distichous to Fibonacci spiral, and then turned into decussate as *Γ* decreased. In the parameter space of EDC2, this order of distribution of major phyllotactic patterns was not affected much by decreasing *A* to moderate values; however, when *A* was further decreased, the orixate pattern appeared in the region of the Fibonacci spiral (Fig 9, S10 Fig). As *A* decreased, the range of *Γ* that produced a Fibonacci spiral became wider and the transition zone between the distichous and Fibonacci spiral patterns, where the divergence angle gradually changed from 180° to 137.5°, became narrower (Fig 9). This result indicated that Fibonacci spiral phyllotaxis is more dominant when assuming a delay in the primordial age-dependent increase in the inhibitory power.

## Discussion

Orixate phyllotaxis is a special kind of alternate phyllotaxis with orthogonal tetrastichy resulting from a four-cycle change in the divergence angle in the order of approximately 180°, 90°, −180° (180°), and −90° (270°); this phyllotaxis occurs in a few plant species across distant taxa [29–32]. In the present study, we investigated a possible theoretical framework behind this minor but interesting phyllotaxis on the basis of the inhibitory field models proposed by Douady and Couder [16, 17], which were shown to give a simple and robust explanation for the self-organization process of major phyllotactic patterns by assuming that each existing leaf primordium emits a constant level of inhibitory power against the formation of a new primordium and that its effect decreases with distance from the primordium. Re-examination of the original versions of Douady and Couder’s models (DC1 and DC2) via exhaustive computer simulations revealed that they do not generate the orixate pattern at any parameter condition. The inability of DC models to produce orixate phyllotaxis prompted us to expand them to account for a more comprehensive generation of phyllotactic patterns. In an attempt to modify DC models, we introduced a temporal change in the inhibitory power during primordium development, instead of using a constant inhibitory power. Such changes of the inhibitory power were partly considered in several previous studies. Douady and Couder assessed the effects of “the growth of the element’s size”, which is equivalent to the primordial age-dependent increase in the inhibitory power and found that it stabilizes whorled phyllotactic patterns [17]. Smith et al. assumed in their mathematical model that the inhibitory power of each primordium decays exponentially with age and stated that this decay promoted phyllotactic pattern formation de novo, as well as pattern transition, and allowed the maintenance of patterns for wider ranges of parameters [9]. A DC1-based model equipped with a primordial age-dependent change in the inhibitory power was also used to investigate floral organ arrangement [35, 36]. In these studies, however, temporal changes in the inhibitory power were examined under limited ranges of parameters focusing on particular aspects of phyllotactic patterning, and the possibility of the generation of minor patterns, such as orixate phyllotaxis, was not addressed.

**Fig 11:**
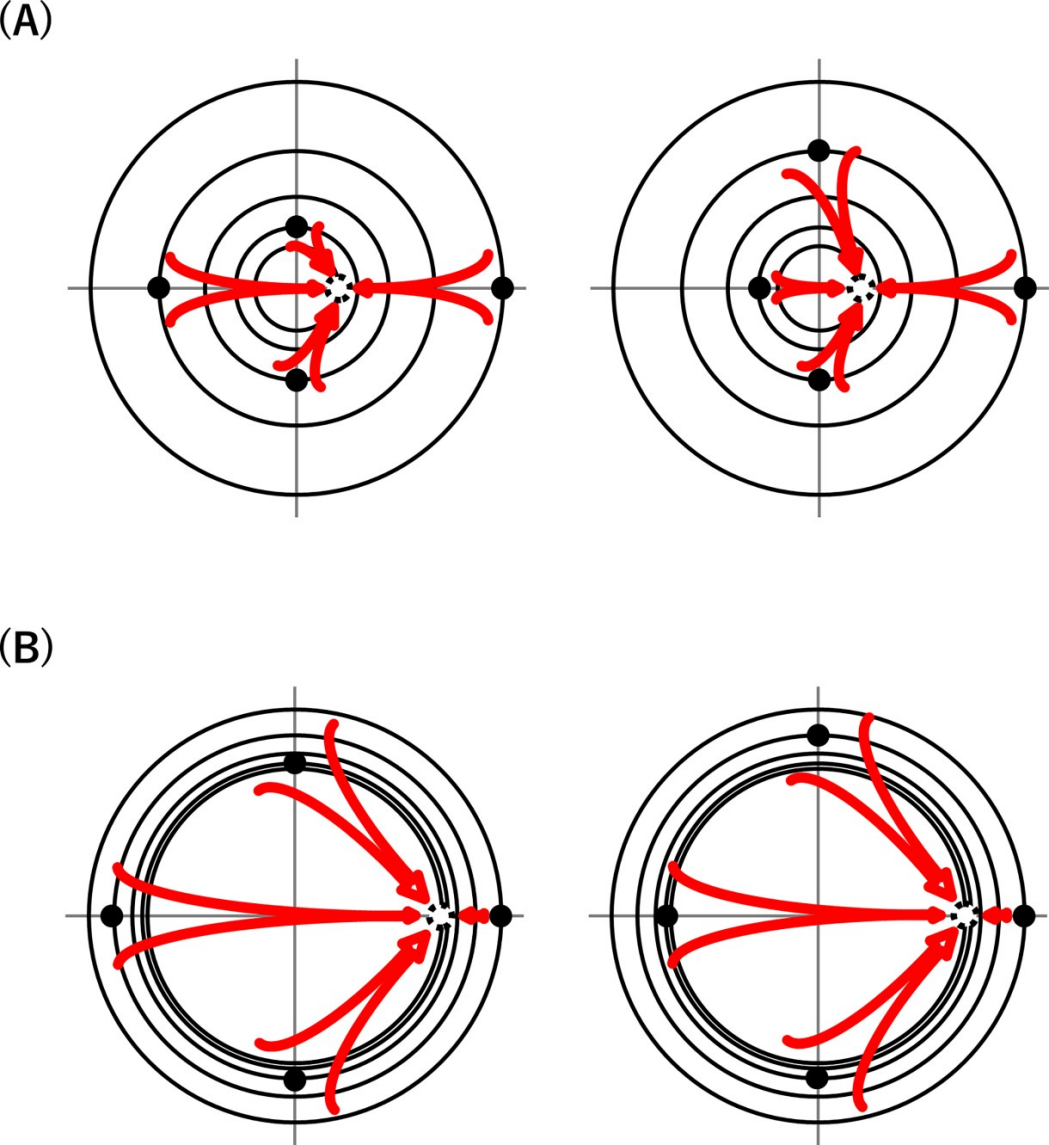
Schematic explanation of two conditions that enable orixate phyllotaxis formation. (A) Gradual increase of the inhibitory power with a relatively small size of SAM. (B) Sudden decrease of the inhibitory power with a relatively large size of SAM. EDC1 can establish orixate phyllotaxis under either of these conditions while EDC2 can only under the former condition.

We expanded DC1 into EDC1 and DC2 into EDC2 by simply incorporating the assumption that the inhibitory power of a primordium is not necessarily constant but may increase or decrease sigmoidally with its age. Extensive computer simulations performed using EDC1 and EDC2 over wide ranges of parameters demonstrated that both of the expanded models can produce orixate phyllotaxis under some parameter conditions. In EDC1, orixate patterns occurred when the inhibitory power was set to increase gradually at large values of the parameter *G*, which represent a small SAM relative to the growth velocity and/or plastochron, and when the inhibitory power decreased suddenly after a certain time lag of about 3*T* at small *G* values, which represent a large SAM. In these two conditions, orixate phyllotactic patterns obviously arise for distinct reasons (Fig 11). Here, let us consider the effect of four pre-existing primordia, which are arranged in the normal orixate pattern on the orthogonal tetrastichy lines, on a new primordium arising at 0°. The key requirements for the formation of a new primordium at 0° to maintain the orixate pattern are: that the inhibitory effects of the primordia at ±90° (previous and second or third previous primordia) are balanced at the site of new primordium formation, and that the inhibitory effect from the fourth previous primordium at 0° is negligible. In the case of a large *G* value with a gradual increase in the inhibitory power, the primordia at ±90° are quite different in the distance to the new primordium site, but their effect can be equalized because of the compensation of the distance-dependent decrease in the inhibitory effect by the age-dependent increase in the inhibitory power, and the fourth previous primordium has little impact because it is located far away. In contrast, in the case of a small *G* value with a sudden decrease in the inhibitory power, the primordia at ±90° exhibit almost the same distance and, therefore, almost the same strength of influence on the site of formation of the new primordium, and the fourth previous primordium no longer has an impact because of the immediately preceding sharp drop in its inhibitory power.

In EDC2, the constraint imposed in EDC1 that leaf primordia are formed one by one at a regular time interval is removed, which allows the simultaneous formation of two or more primordia. Probably because the removal of this constraint destabilizes orixate patterning with a sudden decrease in the inhibitory power, EDC2, unlike EDC1, generated orixate phyllotaxis as a stable pattern only when the inhibitory power was assumed to increase at a late stage and slowly. The orixate patterns produced using EDC2 under this condition had relatively small and large plastochron ratios for the opposite and adjacent pairs of primordia, respectively. A similar feature was observed in the phyllotactic pattern of the winter buds of *O*. *japonica* and was previously reported for the orixate phyllotactic patterns of *Kniphofia* [32]. These findings suggest that orixate pattern generation in computer simulations performed using EDC2 reflects actual phyllotaxis development and that the occurrence of orixate phyllotaxis in distant plant species can be generally explained by the slow and late increase in the inhibitory power. In real plants, the first leaf primordium arises under some influence of pre-existing structures such as cotyledons, which should be considered as the initial condition in model simulation analysis. However, as simulations with EDC2 under two different initial conditions produced orixate patterns at similar parameter settings, orixate phyllotaxis seems not to require specific initial conditions.

There are two views regarding the relationship between orixate phyllotaxis and major phyllotactic patterns. One view was derived from ontogenic observations and regards orixate phyllotaxis as an intermediate form between the distichous and decussate patterns [29], while the other view was derived from a theoretical consideration of symmetry-breaking processes and regards orixate phyllotaxis as an intermediate form between the spiral and decussate patterns [37]. In the parameter space of EDC2, orixate patterns were located in the vicinities of the regions of the decussate, Fibonacci spiral, and Lucas spiral patterns, which indicates a close relationship between orixate phyllotaxis and the decussate and spiral patterns, but not the distichous phyllotaxis; thus, this observation favors the latter view. Among the neighbors of orixate phyllotaxis, oscillating patterns were also found, including a semi-decussate-like one, which could not be generated in DC2. Semi-decussate or semi-decussate-like phyllotaxis is quite rare in nature and has been described in only a few plants, such as *Dioscorea sansibarensis*, *Najas guadalupensis*, and *Kniphofia* “Tubergeniana” [30–32]. The tomato plant (*Solanum lycopersicum*) Shin-Toyotama No. 2, a Japanese cultivar, and *e-2*, a mutant of *Sister-of-PIN1*, which is a paralogue of the auxin-efflux carrier gene *PIN1*, were also reported to exhibit a semi-decussate pattern [38, 39]. Among these plants, *K*. “Tubergeniana” is of particular interest, because its relatives of the same genus have orixate phyllotaxis (*K*. *uvaria*, *K*. *pumila*, and *K*. *tysonii*) or spiral phyllotaxis (*K*. *northiae*) [30, 32]. This phyllotactic variety in *Kniphofia* fits well the simulation result that the spiral and semi-decussate-like patterns were located close to the orixate pattern in the EDC2 parameter space and can be converted into the orixate pattern by small changes in the parameters.

The Fibonacci spiral with a divergence angle close to the golden angle (137.5°) is one of the most common patterns of phyllotaxis observed in plants and is predominant among the spiral phyllotactic patterns. Although this pattern can be generated by previous inhibitory field models, such as DC models, its dominance has not been fully explained by these models [40]. For example, in DC2, the divergence angle of alternate phyllotaxis is shifted gradually from 180° (distichous) to 137.5° (Fibonacci spiral) as the parameter *Γ* is reduced from 2.6 to 1.9 at *α* = 8 and *N* = 1/3, and the range of *Γ* that generates the Fibonacci spiral is not wider than that observed for the other spirals [17]. Our computer simulations performed using EDC2 showed that, compared with DC2, the expanded model assigns a smaller area to spiral patterns with a non-golden angle in the parameter space. This tendency in EDC2 suggests that the dominant occurrence of the golden spiral in nature may be better explained by introducing primordial age-dependent changes in the inhibitory power into the inhibitory field model. In summary, we here propose EDC2 as a most appropriate abstract model of phyllotaxis that can generate a wide range of phyllotactic patterns, including not only major types but also minor types of phyllotaxis, with reasonable proportions comparable to the frequencies of their natural occurrence.

At the molecular level, phyllotactic patterning is now believed to be based on the regulation of the PIN1-driven polar transport of auxin [28]. According to the widely accepted auxin-transport-based model, the membrane localization of PIN1 in the epidermis of the shoot apical region is regulated in response to auxin concentrations in neighboring cells, to build up the auxin gradient, which forms a positive feedback loop that results in the spontaneous establishment of auxin convergence, leading to primordium initiation. In this framework, the age-dependent increase in the inhibitory power included in EDC models is supposed to reflect that the range at which the auxin convergence absorbs auxin expands with time after its emergence. A possibly relevant assumption was included in Smith et al.’s auxin model [25], in which, in addition to the positive feedback dynamics between the auxin gradient and PIN1 localization, it is assumed that PIN1 proteins of all epidermal cells of each primordium are polarized to the tip of the primordium and that this polarization is maintained throughout the growth of the primordium. We now hypothesize that, following the establishment of auxin convergence by the basic feedback dynamics, the range of auxin polar transport toward the convergence point expands by a mechanism different from the basic dynamics as a primordium develops at the auxin convergence, which may correspond to the primordial age-dependent increase of the inhibitory power in EDC2, and that the timing and rate of this expansion can be greatly different among plant species, which should affect phyllotactic patterning as one of critical determinants. Future investigation of the regulatory properties of auxin polar transport during primordium development would provide clues regarding the molecular mechanisms underlying the presumptive age-dependent increase in the primordial inhibitory power and contribute to understanding the variation and limitation of phyllotactic patterns.

## Supporting information

S1 Text Mathematical analysis of the stability of the normal orixate phyllotactic pattern in DC1. (DOCX)

S1 FigMathematical analysis of the conditions required for normal orixate phyllotaxis in EDC1.(A) Two different situations of the arrangement of the four preceding primordia, *L*_*n*−4_, *L*_*n*−3_, *L*_*n*−2_, and *L*_*n*−1_, relative to the incipient primordium *L*_*n*_ in normal orixate phyllotaxis. (B) The blue and red curves show numerical solutions of ${\frac{dI(\theta )}{d\theta }|}_{\theta -\theta_{n-4i}=0}=0$ in situations 1 and 2, respectively. Their intersection points are expected to give the parameter conditions of EDC1 that are required for stabilizing the normal orixate phyllotaxis. (C) Inhibitory field strength on the periphery of SAM in situation 1 (blue) and situation 2 (red) at the parameter settings determined as solutions of ${\frac{dI(\theta )}{d\theta }|}_{\theta -\theta_{n-4i}=0}=0$ that are common to both of these situations. Graphs were drawn with *θ*_*n*−4*i*_ as 0°.(TIF)

S2 Fig Divergence angles of the tetrastichous alternate patterns generated in computer simulations using EDC1. For tetrastichous alternate patterns with a four-cycle change in the divergence angle generated in computer simulations using EDC1 under the conditions of *G* = 0.1 and *a*<0 (A), *G* = 0.5 and *a*>0 (B), and *G* = 1 and *a*>0 (C), the absolute values of divergence angles were plotted using the larger value as the abscissa and the smaller value as the ordinate, such that a pattern with a divergence angle change in the sequence of *p*, *q*, −*p*, and –*q* (|*p*|>|*q*|) was represented by a black dot at the position (|*p*|,|*q*|). The blue dots show the averages determined from the real data of *P*_1_~*P*_2_ to *P*_6_~*P*_7_ (Fig 4D) for each winter bud of *O*. *japonica*.
(TIF)

S3 Fig Transitions of phyllotactic patterns by a slight increase of *b* in the computer simulation with EDC1. Computer simulations with EDC1 were performed under the parameter condition of *G* = 0.5, *a* = 10, and *b* = 5, 5.1, or 5.2. Changes in the divergence angle from *L*_70_~*L*_71_ to *L*_99_~*L*_100_ are shown for the resultant patterns. A small-angle spiral was obtained at *b* = 5.1 (blue circle), while five-cycle and six-cycle alternate patterns were produced at *b* = 5 and at *b* = 5.2, respectively.
(TIF)

S4 Fig Phyllotactic patterns generated in computer simulations using EDC2 with a broad range of settings of *A*. Computer simulations using EDC2 were performed under various parameter settings (201 settings for −100≤*A*≤100, 101 settings for *B*, and 3 settings for *Γ*), and the patterns obtained are displayed according to the color legend shown in Fig 3. Simulations were started by placing a single primordium on the SAM periphery. *N* was fixed at 1/3.
(TIF)

S5 Fig Computer simulations using EDC2 over a wide range of combinations of five parameters. A computer simulation was performed using EDC2 under various settings of five parameters, 101 settings for *A* (0≤*A*≤20), 101 settings for *B* (0≤*B*≤1), 3 settings for *α* (*α* = 1, 2, or 4), 9 settings for *Γ* (1≤*Γ*≤3), and 2 settings for *N* (*N* = 1/3 or 1). The patterns obtained are displayed in the *AB* space according to the color legend shown in Fig 3. Simulations were started by placing a single primordium or two primordia at the central angle of 120° on the SAM periphery.
(TIF)

S6 Fig Semi-decussate-like patterns generated in computer simulations using EDC2. (A) Computer simulations using EDC2 were performed under various parameter settings (101 settings for 0≤*A*≤20, 101 settings for 0≤*B*≤1, and *Γ* = 2, 2.5, or 3) with fixed parameters *α* = 1 and *N* = 1/3. The patterns obtained were converted into colors according to the color legend (Fig 3), and the areas containing semi-decussate-like patterns (blue asterisks) were cut out from the color diagrams. (B) Contour map of the natural log of the inhibitory field strength *I* within the shoot apical region generating semi-decussate-like phyllotaxis with divergence angles of 171° and 89° in the computer simulation using EDC2 under the indicated parameter condition.
(TIF)

S7 Fig Divergence angles of the tetrastichous alternate patterns generated in computer simulations using EDC2. For tetrastichous alternate patterns with a four-cycle change in the divergence angle generated in computer simulations using EDC2 at *Γ* = 2 (A), *Γ* = 2.5 (B), and *Γ* = 3 (C) under the condition of *α* = 1, *N* = 1/3, and *A*>0, absolute values of divergence angles are plotted using the larger value as the abscissa and the smaller value as the ordinate, such that a pattern with a divergence angle change in the sequence of *p*, *q*, −*p*, and −*q* (|*p*|>|*q*|) is represented by a dot at the position (|*p*|,|*q*|). The blue dots show the averages determined from the real data of *P*_1_~*P*_2_ to *P*_6_~*P*_7_ (Fig 4D) for each winter bud of *O*. *japonica*.
(TIF)

S8 Fig Analysis of the stability of the normal orixate phyllotaxis in EDC2. To analyze the stability of the normal orixate phyllotaxis in EDC2, we arranged primordia artificially in the normal orixate pattern with a four-cycle divergence angle change in the sequence of exactly 180°, 90°, −180°, and −90° and with a standardized plastochron that oscillated between 0.1 and 0.325. We then tested whether the inhibitory field strength could assign the position of a new primordium to maintain the normal orixate pattern in the EDC2 system at the parameter condition (*A* = 4.8, *B* = 0.72, *Γ* = 2.8, *N* = 1/3, *α* = 1), with which EDC2 generated a realistic orixate pattern in computer simulation (Fig 10). (A) Contour maps of the natural log of the inhibitory field strength *I* in the shoot apical region, at which the preceding primordia were artificially arranged in two situations of the normal orixate pattern. (B) The inhibitory field strength on the SAM periphery at the time of formation of the *n*^th^ primordium *L*_*n*_ was calculated for situation 1 (blue) and situation 2 (red). The inhibitory field strength had a minimum close to the threshold at position 0° in both situations, which allows the positioning of a new primordium to maintain the normal orixate pattern.
(TIF)

S9 Fig Characteristics of phyllotactic patterns generated in computer simulations with EDC2 as influenced by the parameter *B*. Computer simulations using EDC2 were performed under 101 settings of *B* (0≤*B*≤1) at *Γ* = 1 or 3, *A* = 4 or 10, and *α* = 1. Divergence angles and plastochron times determined from the last nine leaf primordia (*L*_92_ to *L*_100_) are shown for the patterns obtained with various *B* settings, which represent characteristics of phyllotactic patterns as influenced by the timing of the increase of the inhibitory power.
(TIF)

S10 Fig Characteristics of phyllotactic patterns generated in computer simulations with EDC2 as influenced by the parameter *Γ*. Computer simulations using EDC2 were performed under 101 settings of *Γ* (1≤*Γ*≤3) at *α* = 1 or 4, *A* = 4 or 10, and *A*×*B* = 3. Divergence angles and plastochron times determined from the last nine leaf primordia (*L*_92_ to *L*_100_) are shown for the patterns obtained with various *Γ* settings, which represent characteristics of phyllotactic patterns as influenced by the ratio of the inhibition range to the SAM size.
(TIF)

S1 MovieOrixate pattern generation in the computer simulation using EDC2.Contour map of the natural log of the inhibitory field strength *I* within the shoot apical region showing orixate pattern generation in the computer simulation using EDC2 under the condition of *A* = 5, *B* = 0.74, *α* = 1, and *N* = 1/3 (for the color legend, see Fig 10A). In this simulation, to facilitate stable pattern formation, *Γ* was expediently set to depend on the time after the start of simulation, *t*_*sim*_ [18]:
$$\Gamma (t_{sim})=\frac{\Gamma_{i}+\Gamma_{f}}{2}-\frac{\Gamma_{i}-\Gamma_{f}}{2}\tanh\left(\frac{t_{sim}-t_{i}}{\tau }\right),$$
where *Γ*_*i*_ = 50, *Γ*_*f*_ = 2.8, *t*_*i*_ = 0.2, and *τ* = 0.3.(MOV)

S1 Code Source codes for DC1 and EDC1 simulations as a compressed archive. (ZIP)

S2 Code Source codes for DC2 and EDC2 simulations as a compressed archive. (ZIP)

S1 Dataset Numerical data of the results of computer simulations with DC1. (ZIP)

S2 Dataset Numerical data of the results of computer simulations with EDC1. (ZIP)

S3 Dataset Numerical data of the results of computer simulations with DC2. (ZIP)

S4 Dataset Numerical data of the results of computer simulations with EDC2. (ZIP)
