# Supplementary material for: Mathematical model studies of the comprehensive generation of major and minor phyllotactic patterns in plants with a predominant focus on orixate phyllotaxis
Source: PLoS Comput Biol. 2019 Jun 6;15(6):e1007044. doi: 10.1371/journal.pcbi.1007044 (PMC6553687; doi:10.1371/journal.pcbi.1007044)
Supplement: S1 Text — (DOCX) [file pcbi.1007044.s001.docx]

**Text S1. Mathematical analysis of the stability of the normal orixate phyllotactic pattern in DC1**

In the present section, we considered the stability of normal orixate phyllotaxis, which has ideal periodic repetition of a sequence of divergence angles consisting of exactly $180^{\circ}$, $90^{\circ}$, $-180^{\circ}$, and $-90^{\circ}$.

Mathematical analysis was performed for the DC1 system, in which the radius of the shoot apical meristem $R_{0}$is 1 and $L_{i}$ is the $i$^th^ leaf primordium located at $\left( r_{i}\cos\theta_{i},r_{i}\sin\theta_{i} \right)$ with $r_{i}>1$. The $L_{i}$’s inhibitory effect $E\left( x \right)$ at $\left( \cos\theta,\sin\theta\right)$ on the SAM periphery is dependent solely on $d_{i}(\theta)$, the distance from $L_{i}$. When the $n$^th^ primordium $L_{n}$ is arising, the inhibitory field strength $I\left( \theta\right)$ at the position $\left( \cos\theta,\sin\theta\right)$ is calculated by summing the inhibitory effects from all existing primordia, as follows.

$$\begin{aligned} I\left( \theta\right)=\sum_{k=1}^{n-1} E\left( d_{k}\left( \theta\right) \right)=\sum_{j=0}^{3} \sum_{i=1}^{\left\lfloor\frac{n-1+j}{4} \right\rfloor} E\left( d_{n-4i+j}\left( \theta\right) \right)\#\left( S1 \right) \end{aligned}$$

When the normal pattern of orixate phyllotaxis is stably maintained, the inhibitory field strength should give a minimum at $\theta=\theta_{n-4i}$. Hence, when setting $\theta_{n-4i}=0$, the following equation should be satisfied:

$$\begin{aligned} \left. \frac{dI\left( \theta\right)}{d\theta} \right|_{\theta=0}=0\#\left( S2 \right) \end{aligned}$$

Because $d_{k}\left( \theta\right)=\sqrt{r_{k}^{2}+1-2r_{k}\cos\left( \theta-\theta_{k} \right)}$, we obtain:

$$\begin{aligned} \left. \frac{dd_{k}\left( \theta\right)}{d\theta} \right|_{\theta=0}=\left\{ \begin{aligned} 0 \left( \theta_{k}=0, \pi\right) \\ \mp\frac{r_{k}}{\varrho\left( r_{k} \right)} \left( \theta_{k}=\pm\frac{\pi}{2} \right) \end{aligned} \right.\#\left( S3 \right) \end{aligned}$$

$$\begin{aligned} d_{k}\left( 0 \right)=\left\{ \begin{aligned} r_{k}-1 \left( \theta_{k}=0 \right) \\ r_{k}+1 \left( \theta_{k}= \pi\right) \\ \varrho\left( r_{k} \right) \left( \theta_{k}=\pm\frac{\pi}{2} \right) \end{aligned} \right.,\#\left( S4 \right) \end{aligned}$$

where $\varrho\left( r \right)\equiv\sqrt{r^{2}+1}$.

Thus,

$$\begin{aligned} \left. \frac{dE\left( d_{k}\left( \theta\right) \right)}{d\theta} \right|_{\theta=0}=\left. \frac{dE\left( x \right)}{dx} \right|_{x=d_{k}\left( 0 \right)}\left. \frac{dd_{k}\left( \theta\right)}{d\theta} \right|_{\theta=0}=\left\{ \begin{aligned} 0 \left( \theta_{k}=0, \pi\right) \\ \mp f\left( r_{k} \right) \left( \theta_{k}=\pm\frac{\pi}{2} \right) \end{aligned} \right.,\#\left( S5 \right) \end{aligned}$$

where $f\left( r \right)\equiv\frac{r}{\varrho\left( r \right)}\left. \frac{dE\left( x \right)}{dx} \right|_{x=\varrho\left( r \right)}$.

Regarding the arrangement of primordia, there are two geometrical situations; in situation 1, the divergence angle between the newly arising primordium, $L_{n}$, and the last primordium, $L_{n-1}$, is $\pm90^{\circ}$ ($\pm\pi/2$), while it is $180^{\circ}$ ($\pi$) in situation 2 (Fig S1A).

(Situation 1)

Situation 1 is represented by setting $\theta_{n-4i+j}$ as:

$$\begin{aligned} \theta_{n-4i+j}=\left\{ \begin{aligned} 0 \left( j=0 \right) \\ \pi\left( j=1 \right) \\ -\frac{\pi}{2} \left( j=2 \right) \\ \frac{\pi}{2} \left( j=3 \right) \end{aligned} \right..\#\left( S6 \right) \end{aligned}$$

The application of this condition to Eq S5 yields:

$$\begin{aligned} \left. \frac{dE\left( d_{n-4i+j}\left( \theta\right) \right)}{d\theta} \right|_{\theta=0}=\left\{ \begin{aligned} 0 \left( j=0, 1 \right) \\ f\left( r_{n-4i+j} \right) \left( j=2 \right) \\ -f\left( r_{n-4i+j} \right) \left( j=3 \right) \end{aligned} \right..\#\left( S7 \right) \end{aligned}$$

Hence,

$$\begin{aligned} \left. \frac{dI\left( \theta\right)}{d\theta} \right|_{\theta=0}=\sum_{j=0}^{3} \sum_{i=1}^{\left\lfloor\frac{n-1+j}{4} \right\rfloor} \left. \frac{dE\left( d_{n-4i+j}\left( \theta\right) \right)}{d\theta} \right|_{\theta=0} \\ =\sum_{i=1}^{\left\lfloor\frac{n+1}{4} \right\rfloor} f\left( r_{n-4i+2} \right)-\sum_{i=1}^{\left\lfloor\frac{n+2}{4} \right\rfloor} f\left( r_{n-4i+3} \right).\#\left( S8 \right) \end{aligned}$$

Because $E\left( x \right)$ is a monotonically decreasing function,$f\left( r \right)$ is always negative:

$$\begin{aligned} \left. \frac{dI\left( \theta\right)}{d\theta} \right|_{\theta=0}\geq\sum_{i=1}^{\left\lfloor\frac{n+1}{4} \right\rfloor} \left\{ f\left( r_{n-4i+2} \right)-f\left( r_{n-4i+3} \right) \right\}.\#\left( S9 \right) \end{aligned}$$

(Situation 2)

Situation 2 is represented by setting $\theta_{n-4i+j}$ as:

$$\begin{aligned} \theta_{n-4i+j}=\left\{ \begin{aligned} 0 \left( j=0 \right) \\ -\frac{\pi}{2} \left( j=1 \right) \\ \frac{\pi}{2} \left( j=2 \right) \\ \pi\left( j=3 \right) \end{aligned} \right..\#\left( S10 \right) \end{aligned}$$

The $\theta$-derivative of $I\left( \theta\right)$ can be calculated as in the case described for situation 1:

$$\begin{aligned} \left. \frac{dI\left( \theta\right)}{d\theta} \right|_{\theta=0}=\sum_{i=1}^{\left\lfloor\frac{n}{4} \right\rfloor} f\left( r_{n-4i+1} \right)-\sum_{i=1}^{\left\lfloor\frac{n+1}{4} \right\rfloor} f\left( r_{n-4i+2} \right) \\ \geq\sum_{i=1}^{\left\lfloor\frac{n}{4} \right\rfloor} \left\{ f\left( r_{n-4i+1} \right)-f\left( r_{n-4i+2} \right) \right\}.\#\left( S11 \right) \end{aligned}$$

According to the distance dependency of the inhibitory effect assumed in DC1, $E\left( \varrho\right)=k\varrho^{-\eta}$. Using this assumption and noting that $\eta>0$ and $r>1$, we obtain:

$$\begin{aligned} \frac{df\left( r \right)}{dr}=\frac{d}{dr}\left( \frac{r}{\varrho}\frac{d}{d\varrho}k\varrho^{-\eta} \right)=k\eta\varrho^{-\eta-4}\left\{ \left( \eta+1 \right)r^{2}-1 \right\}>0.\#\left( S12 \right) \end{aligned}$$

As $f\left( r \right)$ increases monotonically with $r$, $f\left( r_{n-4i+1} \right)>f\left( r_{n-4i+2} \right)>f\left( r_{n-4i+3} \right)$, and then $\left. \frac{dI\left( \theta\right)}{d\theta} \right|_{\theta=0}>0$ in both situations. This indicates that the total inhibitory field strength cannot satisfy Eq S2, which demonstrates that normal orixate phyllotaxis cannot be established in DC1.
