# Supplementary figures and images for: Mathematical model studies of the comprehensive generation of major and minor phyllotactic patterns in plants with a predominant focus on orixate phyllotaxis
Source: PLoS Comput Biol. 2019 Jun 6;15(6):e1007044. doi: 10.1371/journal.pcbi.1007044 (PMC6553687; doi:10.1371/journal.pcbi.1007044)

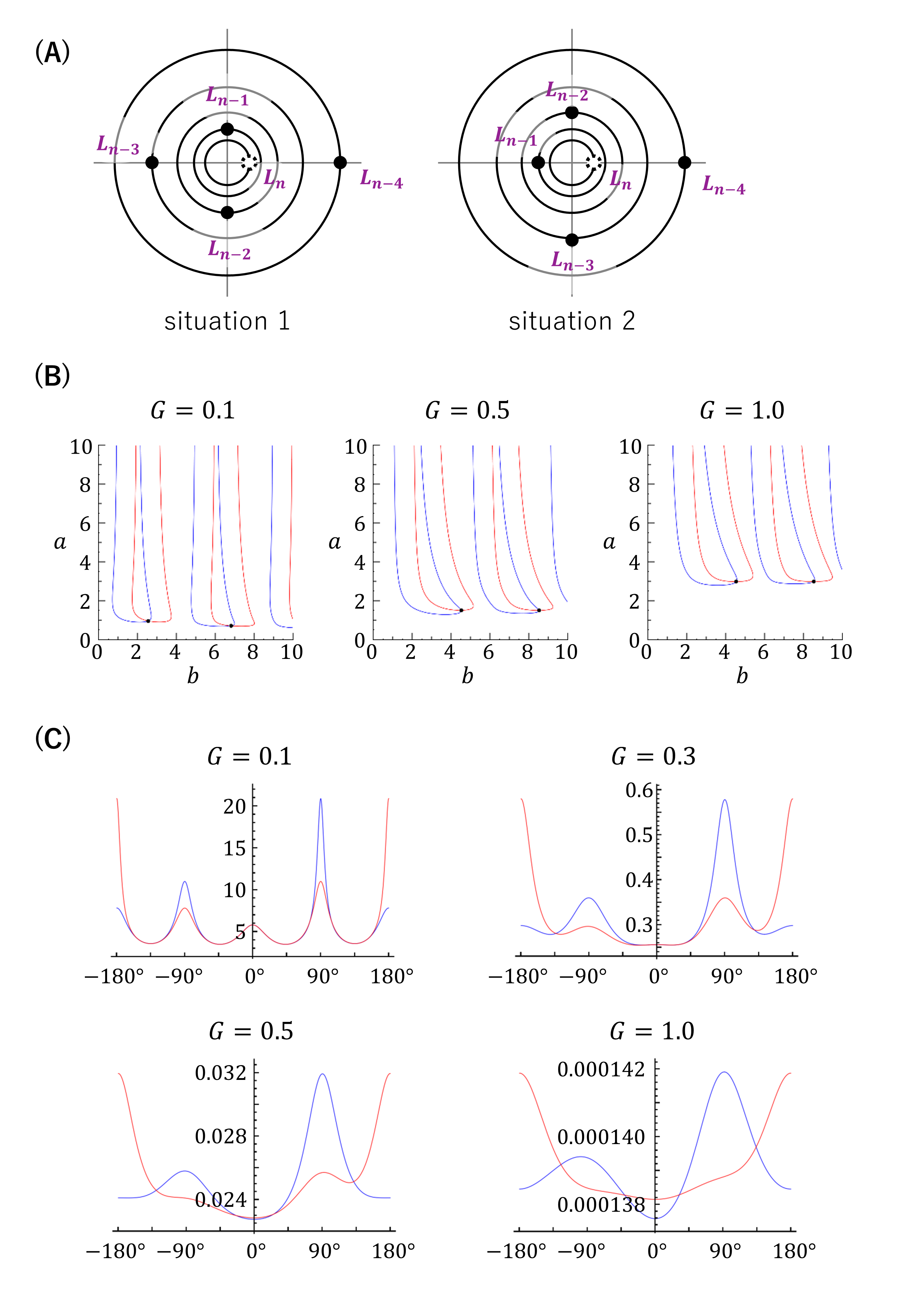

Supplement: S1 Fig — (A) Two different situations of the arrangement of the four preceding primordia, Ln−4, Ln−3, Ln−2, and Ln−1, relative to the incipient primordium Ln in normal orixate phyllotaxis. (B) The blue and red curves show numerical solutions of dI(θ)dθ|θ−θn−4i=0=0 in situations 1 and 2, respectively. Their intersection points are expected to give the parameter conditions of EDC1 that are required for stabilizing the normal orixate phyllotaxis. (C) Inhibitory field strength on the periphery of SAM in situation 1 (blue) and situation 2 (red) at the parameter settings determined as solutions of dI(θ)dθ|θ−θn−4i=0=0 that are common to both of these situations. Graphs were drawn with θn−4i as 0°. (TIF) [file pcbi.1007044.s002.tif]

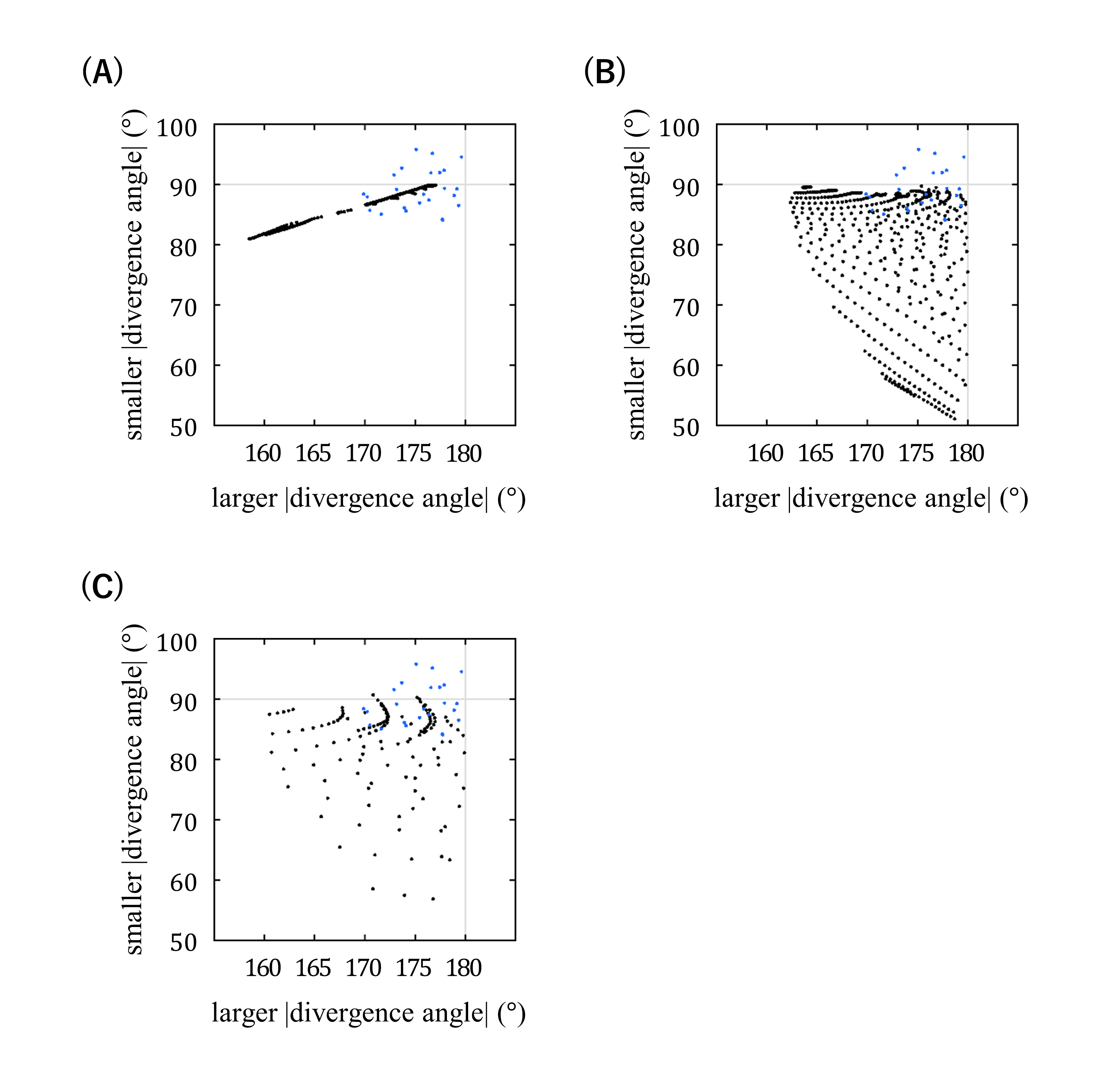

Supplement: S2 Fig — For tetrastichous alternate patterns with a four-cycle change in the divergence angle generated in computer simulations using EDC1 under the conditions of G = 0.1 and a<0 (A), G = 0.5 and a>0 (B), and G = 1 and a>0 (C), the absolute values of divergence angles were plotted using the larger value as the abscissa and the smaller value as the ordinate, such that a pattern with a divergence angle change in the sequence of p, q, −p, and –q (|p|>|q|) was represented by a black dot at the position (|p|,|q|). The blue dots show the averages determined from the real data of P1~P2 to P6~P7 (Fig 4D) for each winter bud of O. japonica. (TIF) [file pcbi.1007044.s003.tif]

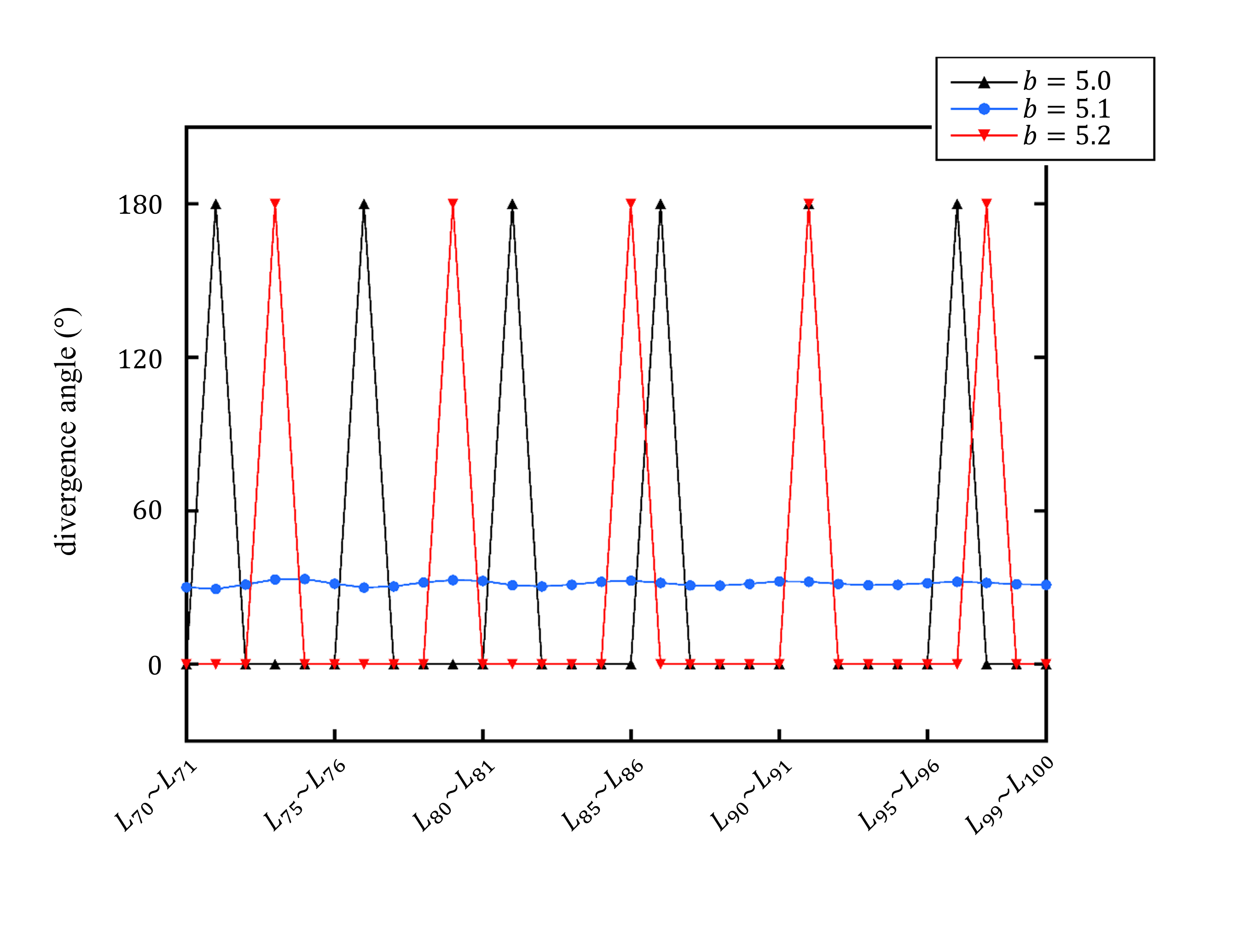

Supplement: S3 Fig — Computer simulations with EDC1 were performed under the parameter condition of G = 0.5, a = 10, and b = 5, 5.1, or 5.2. Changes in the divergence angle from L70~L71 to L99~L100 are shown for the resultant patterns. A small-angle spiral was obtained at b = 5.1 (blue circle), while five-cycle and six-cycle alternate patterns were produced at b = 5 and at b = 5.2, respectively. (TIF) [file pcbi.1007044.s004.tif]

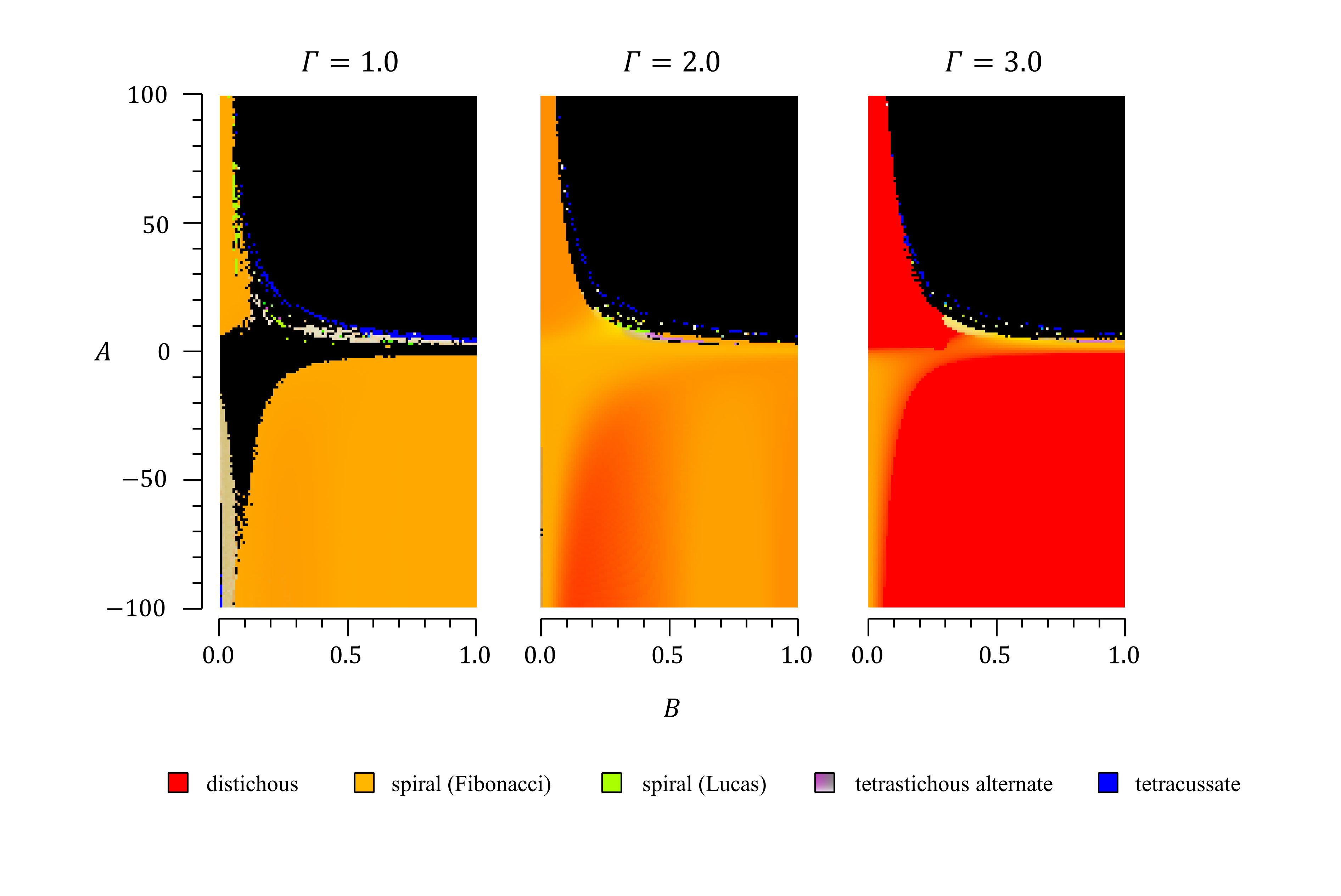

Supplement: S4 Fig — Computer simulations using EDC2 were performed under various parameter settings (201 settings for −100≤A≤100, 101 settings for B, and 3 settings for Γ), and the patterns obtained are displayed according to the color legend shown in Fig 3. Simulations were started by placing a single primordium on the SAM periphery. N was fixed at 1/3. (TIF) [file pcbi.1007044.s005.tif]

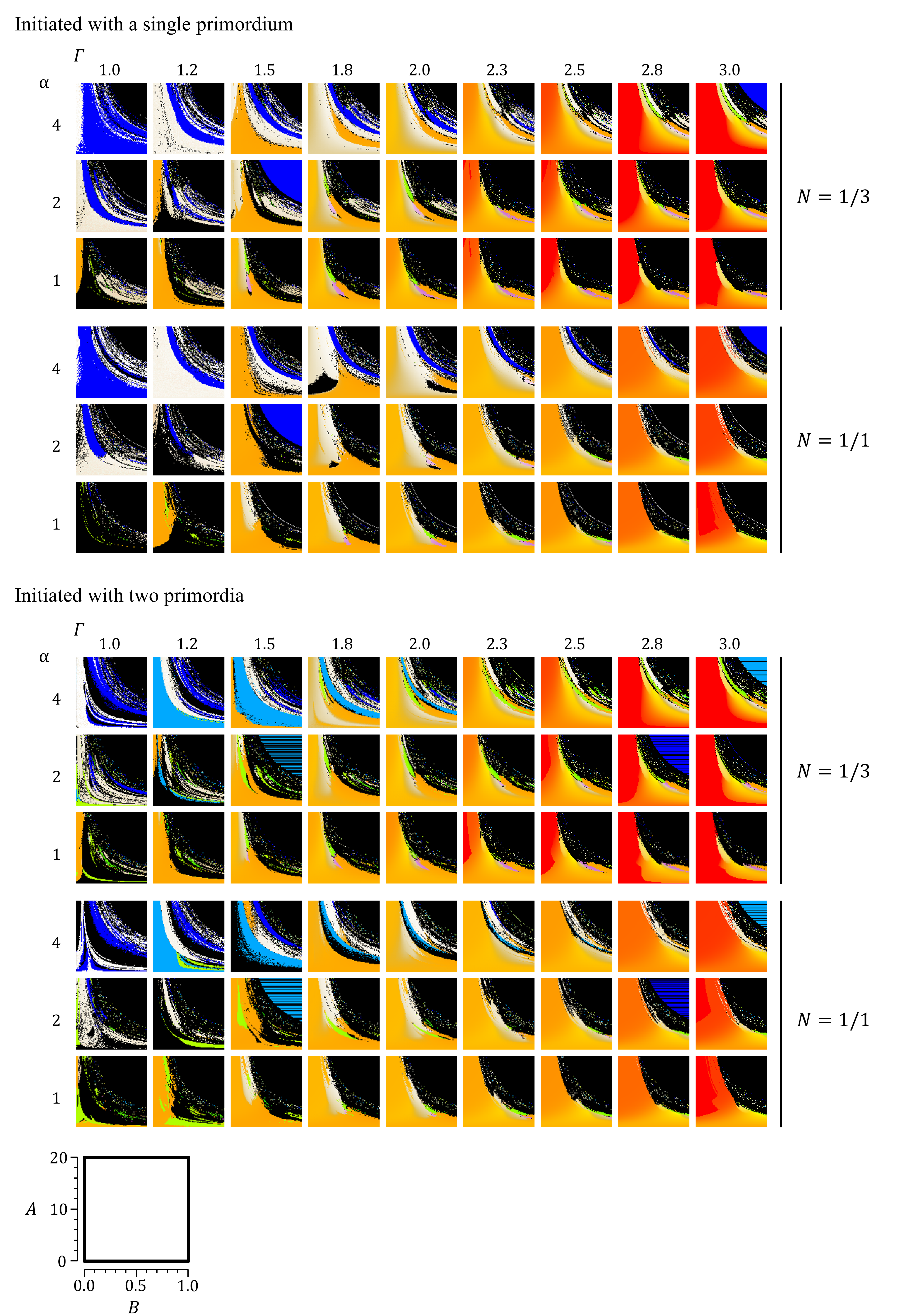

Supplement: S5 Fig — A computer simulation was performed using EDC2 under various settings of five parameters, 101 settings for A (0≤A≤20), 101 settings for B (0≤B≤1), 3 settings for α (α = 1, 2, or 4), 9 settings for Γ (1≤Γ≤3), and 2 settings for N (N = 1/3 or 1). The patterns obtained are displayed in the AB space according to the color legend shown in Fig 3. Simulations were started by placing a single primordium or two primordia at the central angle of 120° on the SAM periphery. (TIF) [file pcbi.1007044.s006.tif]

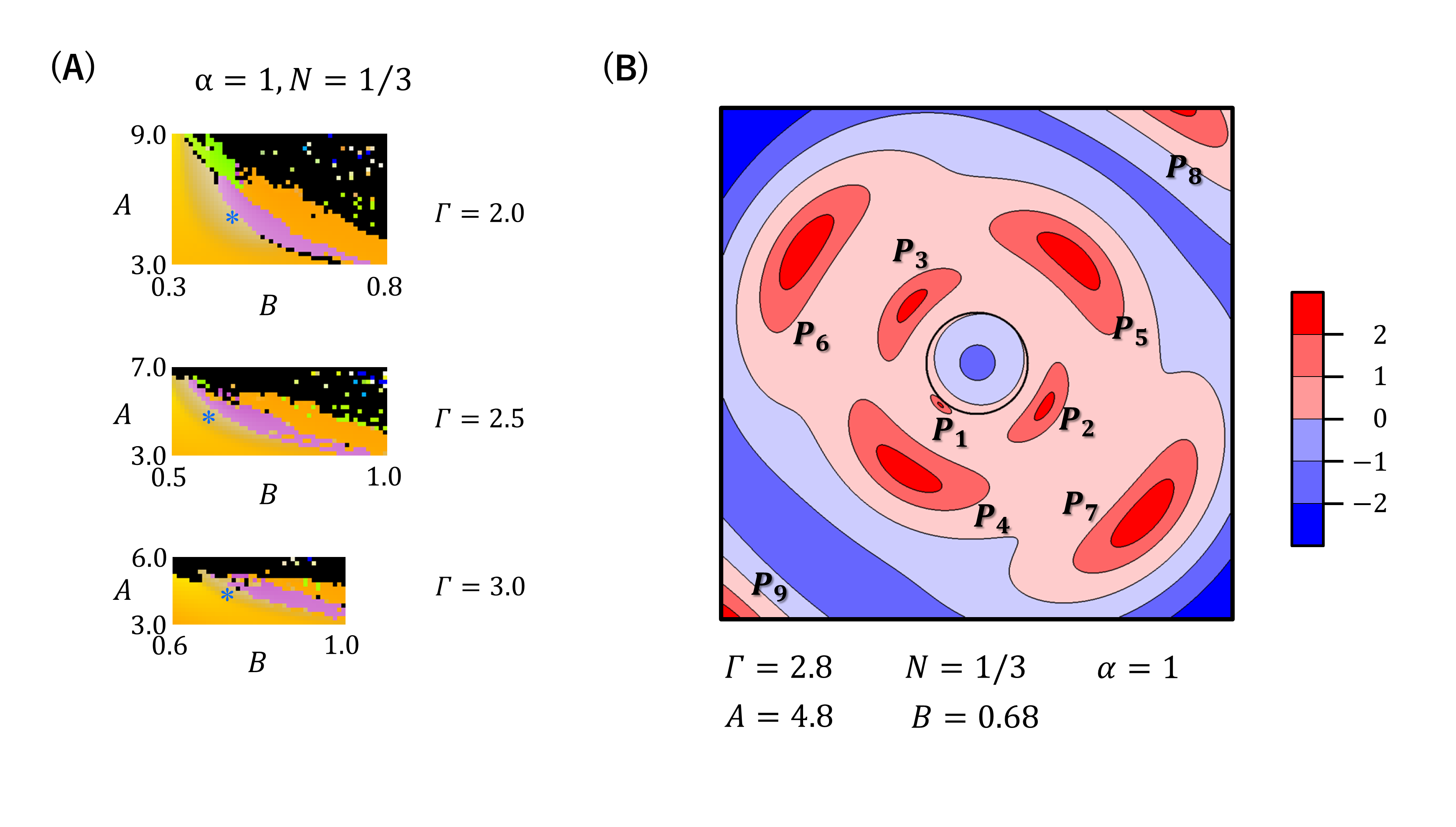

Supplement: S6 Fig — (A) Computer simulations using EDC2 were performed under various parameter settings (101 settings for 0≤A≤20, 101 settings for 0≤B≤1, and Γ = 2, 2.5, or 3) with fixed parameters α = 1 and N = 1/3. The patterns obtained were converted into colors according to the color legend (Fig 3), and the areas containing semi-decussate-like patterns (blue asterisks) were cut out from the color diagrams. (B) Contour map of the natural log of the inhibitory field strength I within the shoot apical region generating semi-decussate-like phyllotaxis with divergence angles of 171° and 89° in the computer simulation using EDC2 under the indicated parameter condition. (TIF) [file pcbi.1007044.s007.tif]

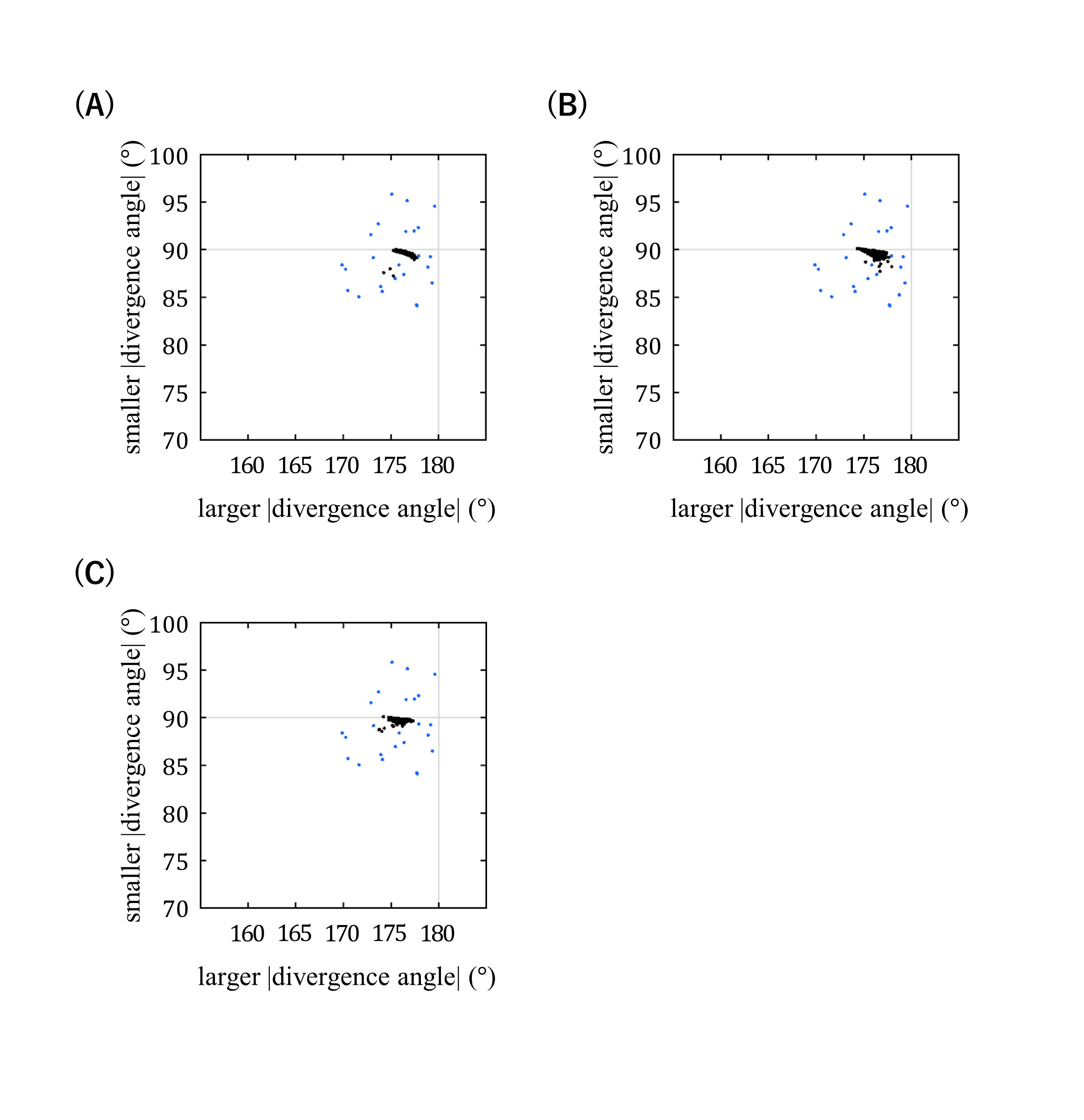

Supplement: S7 Fig — For tetrastichous alternate patterns with a four-cycle change in the divergence angle generated in computer simulations using EDC2 at Γ = 2 (A), Γ = 2.5 (B), and Γ = 3 (C) under the condition of α = 1, N = 1/3, and A>0, absolute values of divergence angles are plotted using the larger value as the abscissa and the smaller value as the ordinate, such that a pattern with a divergence angle change in the sequence of p, q, −p, and −q (|p|>|q|) is represented by a dot at the position (|p|,|q|). The blue dots show the averages determined from the real data of P1~P2 to P6~P7 (Fig 4D) for each winter bud of O. japonica. (TIF) [file pcbi.1007044.s008.tif]

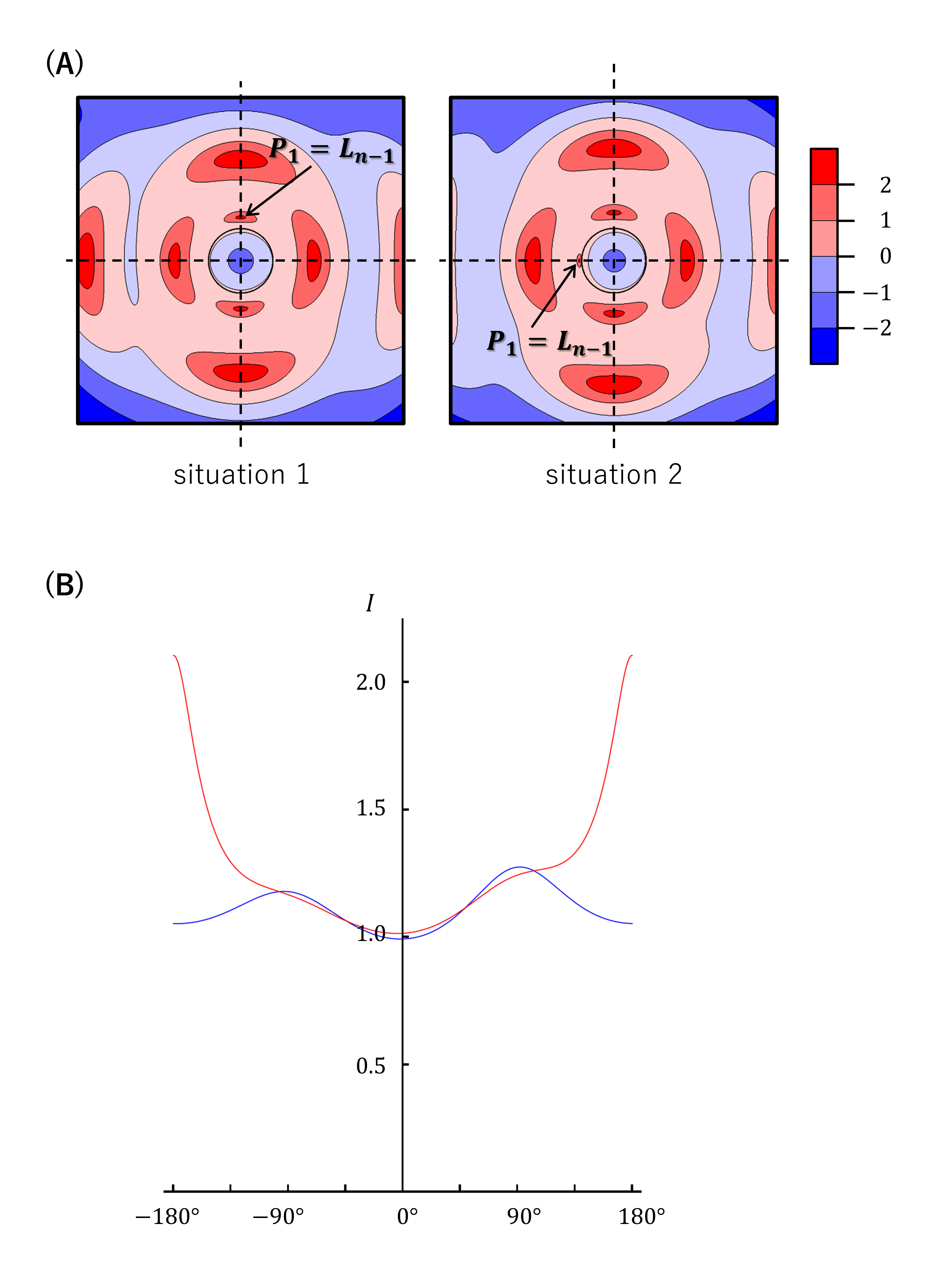

Supplement: S8 Fig — To analyze the stability of the normal orixate phyllotaxis in EDC2, we arranged primordia artificially in the normal orixate pattern with a four-cycle divergence angle change in the sequence of exactly 180°, 90°, −180°, and −90° and with a standardized plastochron that oscillated between 0.1 and 0.325. We then tested whether the inhibitory field strength could assign the position of a new primordium to maintain the normal orixate pattern in the EDC2 system at the parameter condition (A = 4.8, B = 0.72, Γ = 2.8, N = 1/3, α = 1), with which EDC2 generated a realistic orixate pattern in computer simulation (Fig 10). (A) Contour maps of the natural log of the inhibitory field strength I in the shoot apical region, at which the preceding primordia were artificially arranged in two situations of the normal orixate pattern. (B) The inhibitory field strength on the SAM periphery at the time of formation of the nth primordium Ln was calculated for situation 1 (blue) and situation 2 (red). The inhibitory field strength had a minimum close to the threshold at position 0° in both situations, which allows the positioning of a new primordium to maintain the normal orixate pattern. (TIF) [file pcbi.1007044.s009.tif]

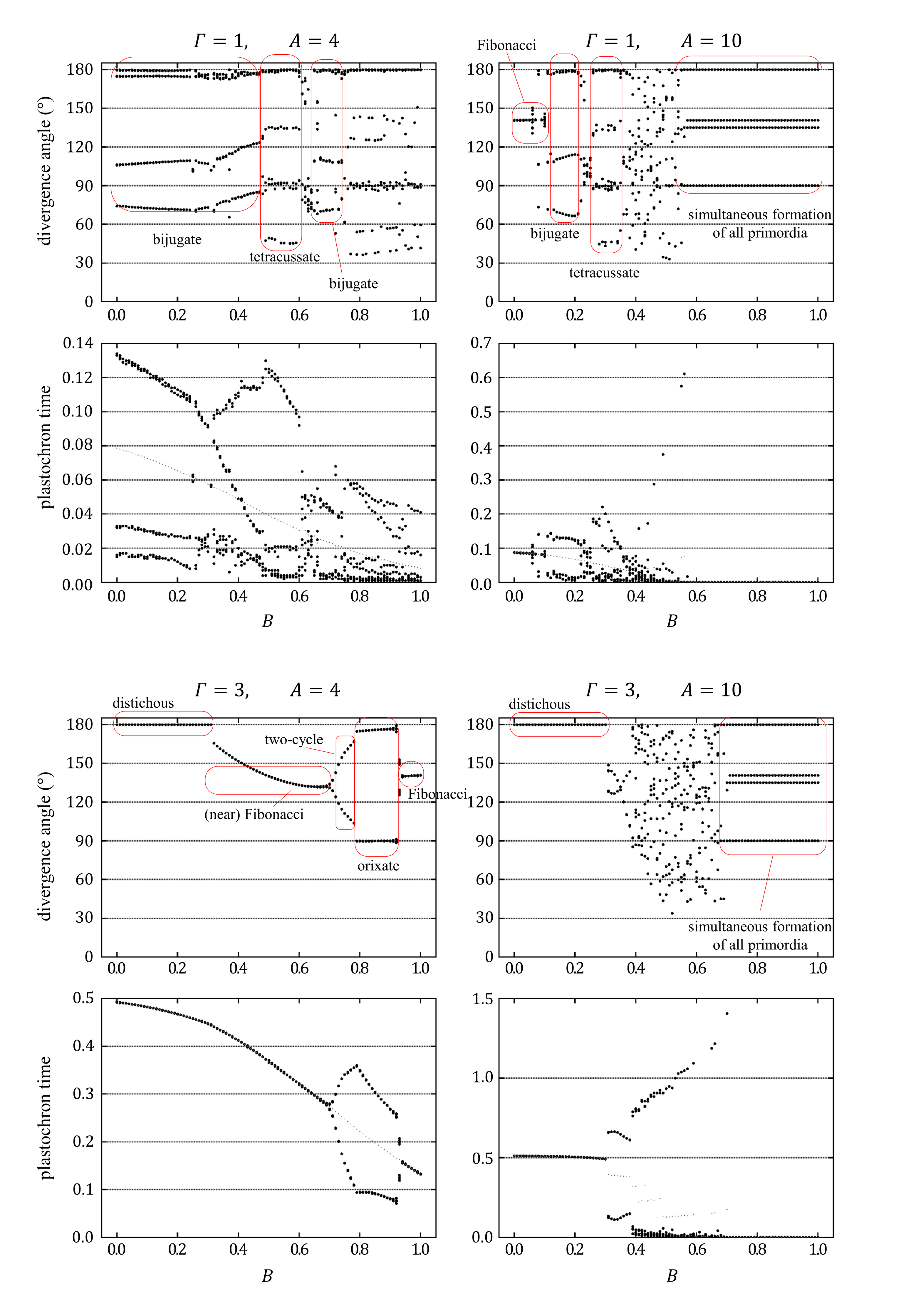

Supplement: S9 Fig — Computer simulations using EDC2 were performed under 101 settings of B (0≤B≤1) at Γ = 1 or 3, A = 4 or 10, and α = 1. Divergence angles and plastochron times determined from the last nine leaf primordia (L92 to L100) are shown for the patterns obtained with various B settings, which represent characteristics of phyllotactic patterns as influenced by the timing of the increase of the inhibitory power. (TIF) [file pcbi.1007044.s010.tif]

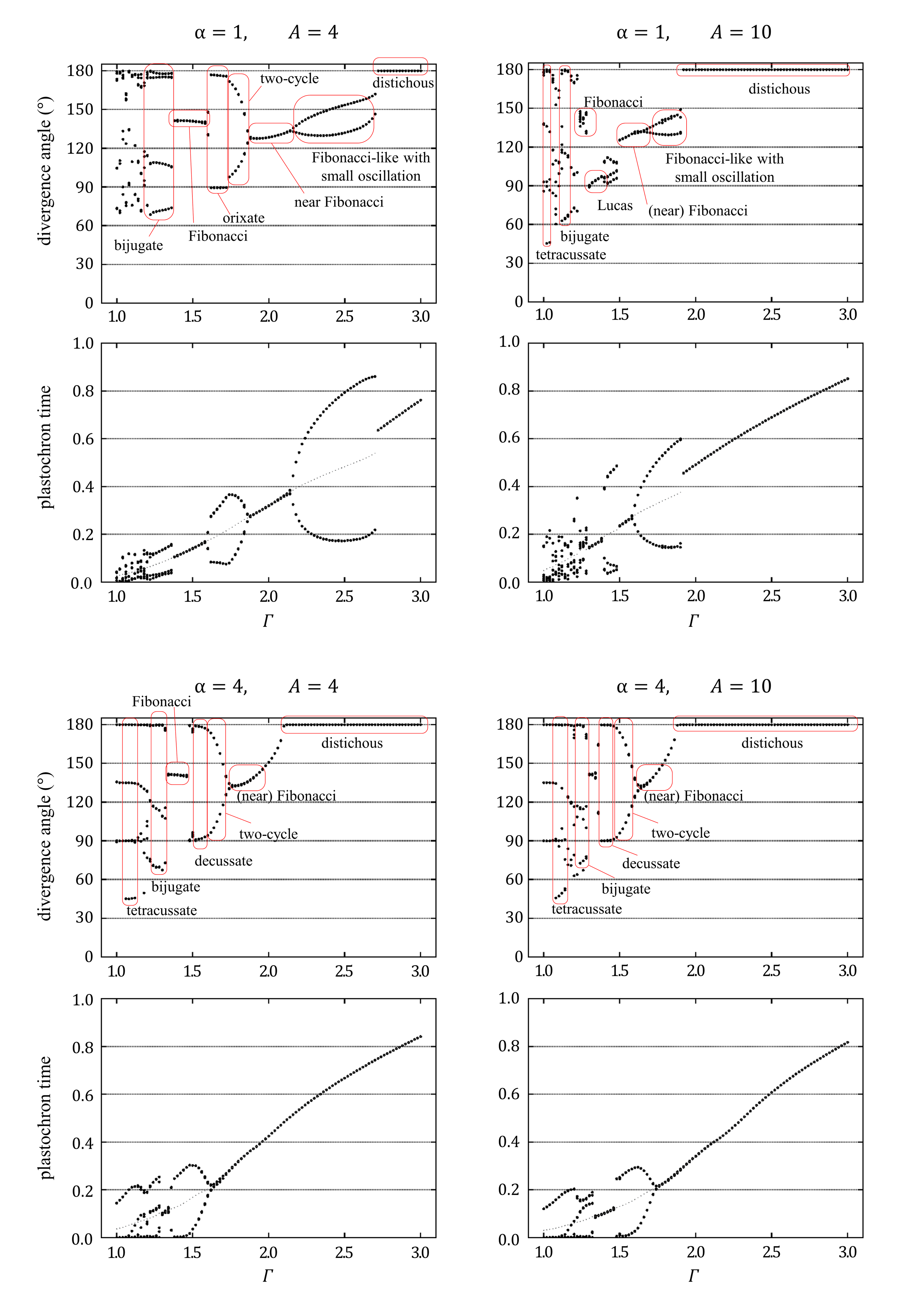

Supplement: S10 Fig — Computer simulations using EDC2 were performed under 101 settings of Γ (1≤Γ≤3) at α = 1 or 4, A = 4 or 10, and A×B = 3. Divergence angles and plastochron times determined from the last nine leaf primordia (L92 to L100) are shown for the patterns obtained with various Γ settings, which represent characteristics of phyllotactic patterns as influenced by the ratio of the inhibition range to the SAM size. (TIF) [file pcbi.1007044.s011.tif]
